# Strategies for promoting neurovascularization in bone regeneration

**DOI:** 10.1186/s40779-025-00596-1

**Published:** 2025-03-03

**Authors:** Xin-Ling Li, Yu-Qing Zhao, Li Miao, Yan-Xin An, Fan Wu, Jin-Yu Han, Jing-Yuan Han, Franklin R. Tay, Zhao Mu, Yang Jiao, Jing Wang

**Affiliations:** 1 State Key Laboratory of Oral & Maxillofacial Reconstruction and Regeneration, National Clinical Research Center for Oral Diseases, Shaanxi Engineering Research Center for Dental Materials and Advanced Manufacture, Department of Oral Implants, School of Stomatology, The Fourth Military Medical University, Xi’an, 710032 China; 2 Department of Stomatology, The Seventh Medical Center of PLA General Hospital, Beijing, 100700 China; 3 Department of General Surgery, The First Affiliated Hospital of Xi’an Medical University, Xi’an, 710077 China; 4 Graduate School of Augusta University, Augusta, GA 30912 USA; 5 State Key Laboratory of Oral & Maxillofacial Reconstruction and Regeneration, School of Stomatology, The Fourth Military Medical University, Xi’an, 710032 China

**Keywords:** Biomaterials, Blood vessels, Bone, Nerve, Neurovascularization

## Abstract

Bone tissue relies on the intricate interplay between blood vessels and nerve fibers, both are essential for many physiological and pathological processes of the skeletal system. Blood vessels provide the necessary oxygen and nutrients to nerve and bone tissues, and remove metabolic waste. Concomitantly, nerve fibers precede blood vessels during growth, promote vascularization, and influence bone cells by secreting neurotransmitters to stimulate osteogenesis. Despite the critical roles of both components, current biomaterials generally focus on enhancing intraosseous blood vessel repair, while often neglecting the contribution of nerves. Understanding the distribution and main functions of blood vessels and nerve fibers in bone is crucial for developing effective biomaterials for bone tissue engineering. This review first explores the anatomy of intraosseous blood vessels and nerve fibers, highlighting their vital roles in bone embryonic development, metabolism, and repair. It covers innovative bone regeneration strategies directed at accelerating the intrabony neurovascular system over the past 10 years. The issues covered included material properties (stiffness, surface topography, pore structures, conductivity, and piezoelectricity) and acellular biological factors [neurotrophins, peptides, ribonucleic acids (RNAs), inorganic ions, and exosomes]. Major challenges encountered by neurovascularized materials during their clinical translation have also been highlighted. Furthermore, the review discusses future research directions and potential developments aimed at producing bone repair materials that more accurately mimic the natural healing processes of bone tissue. This review will serve as a valuable reference for researchers and clinicians in developing novel neurovascularized biomaterials and accelerating their translation into clinical practice. By bridging the gap between experimental research and practical application, these advancements have the potential to transform the treatment of bone defects and significantly improve the quality of life for patients with bone-related conditions.

## Background

Bone is essential to all mammalian species by functioning as a living organ that enables movement. Rehabilitation of bone defects that result from limb trauma, degenerative pathology, or tumor resection poses a challenge for physicians once they exceed a critical size [1–4]. In the world, as many as 2 million bone grafting procedures are performed annually. This number is expected to increase to over 3 million by 2030, as a result of the aging population and the rising prevalence of bone-related conditions [5].

Autologous bone grafts remain the gold standard for the treatment of bone defects because of their high success rate and compatibility with a patient’s own tissue, minimizing the risk of immune rejection. However, significant concerns persist, including the limited availability of donor bone, the need for a secondary surgical procedure to harvest the graft, and potential complications at the donor site, such as chronic pain, pathogenic infection, and structural weakness [6–8]. These issues drive the ongoing search for effective alternative treatments and the development of biomaterials that can mimic the biological and functional properties of natural bone tissue, while reducing the associated risks and limitations [9]. Nevertheless, most synthetic biomaterials only replicate the mechanical properties and macroscopic structures of bone. These biomaterials lack the ability to accurately mimic the complex bone microenvironment and functional units. They often fall short in promoting the same level of osteogenesis, vascularization, and integration with existing bone tissue. As a result, the healing process is slower and less effective. These limitations highlight the need for continued research and development of advanced biomaterials that can more closely emulate the biological and functional characteristics of natural bone.

**Fig. 1:**
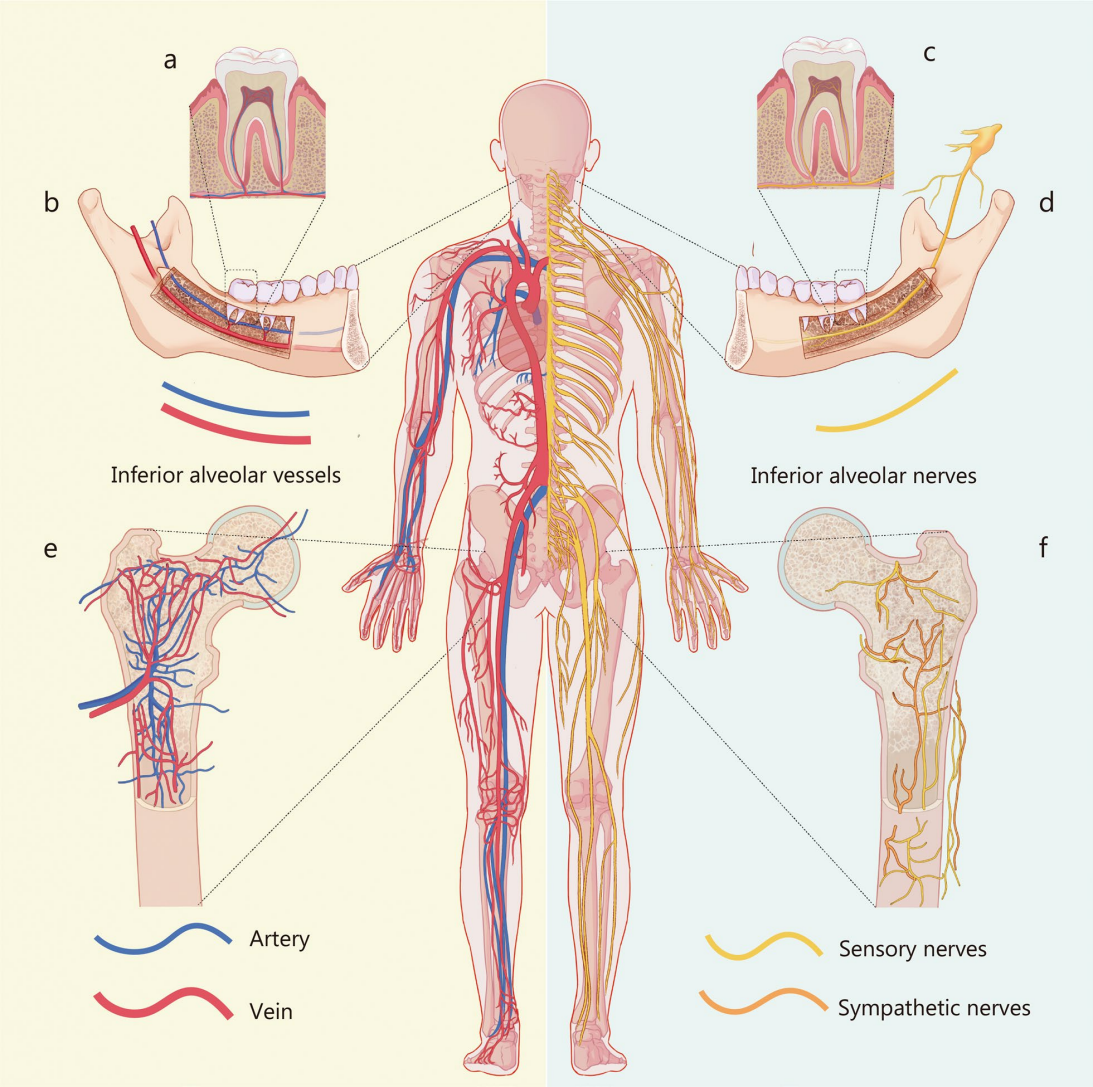
Distribution of blood vessels and nerves in different parts of the body, including the teeth, jaws, and femurs. **a** The inferior alveolar artery and vein within the mandible. **b** The inferior alveolar artery and vein branching out to the pulp chamber of the mandibular molar. **c** The inferior alveolar nerve within the mandible. **d** The inferior alveolar nerve branching out to the pulp chamber of the mandibular molar. **e** Distribution of blood vessels within the femur, which mainly consists of periosteal artery, nutrient artery and emissary vein. **f** Distribution of nerves within the femurs, including sensory nerves and sympathetic nerves

Osteogenesis is not solely the work of bone cells, but involves the collaboration of multiple systems, including the vascular and nervous systems [10–12]. The general distribution of blood vessels and nerves in the body is shown in Fig. 1. The figure illustrates the widespread distribution of blood vessels and nerves in the teeth, jaws, and femurs. Bone cells, including osteoblasts, osteoclasts, and osteocytes, are central to the formation, resorption, remodeling, and maintenance of bone tissue. However, these processes are heavily influenced by the vascular system, which supplies the basic necessities, such as oxygen, nutrients, and growth factors, and provides a pathway to remove the waste products; Blood vessels also play a key role in the recruitment of osteoprogenitor cells to sites of bone formation and repair [13–16]. However, when designing bone repair materials, nerves have often been overlooked [17]. Nerves secrete neurotransmitters, neurotrophins, and neuropeptides that influence bone cell activity and differentiation [18–20]. Moreover, nerves guide angiogenesis and control the blood flow in intrabony blood vessels [21–23]. Consequently, the crosstalk between bone cells, endothelial cells (ECs), nerve cells, and immune cells creates a specific microenvironment to restore homeostasis and regulate tissue repair [24–27]. Many kinds of tissue-specific biomaterials with bioactive components that promote neurovascular regeneration have been developed in order to enhance the therapeutic efficacy. Recent studies have systematically introduced new advances in cellular crosstalk between bone and nervous system [28–32]. Nevertheless, few have comprehensively summarized the regulatory effect of the neurovascular system  in the bone microenvironment.

In this review, the anatomic structures and functions of neurovascular coupling in bone development, metabolism and repair are first introduced. This is followed by a discussion on the design strategy and mechanism of neurovascularized materials to promote osteogenesis. Then, future direction perspectives in this field are proposed to provide a platform for clinical translation. These therapies aim to improve patient outcomes by enhancing the integration and functionality of bone grafts, reducing healing times, and minimizing complications. By bridging the gap between experimental research and practical applications, these advancements have the potential to fundamentally revolutionize the treatment of bone defects, and to improve the quality of life for patients suffering from bone-related conditions.

## Neurovascular coupling in bone development, metabolism and repair

**Fig. 2:**
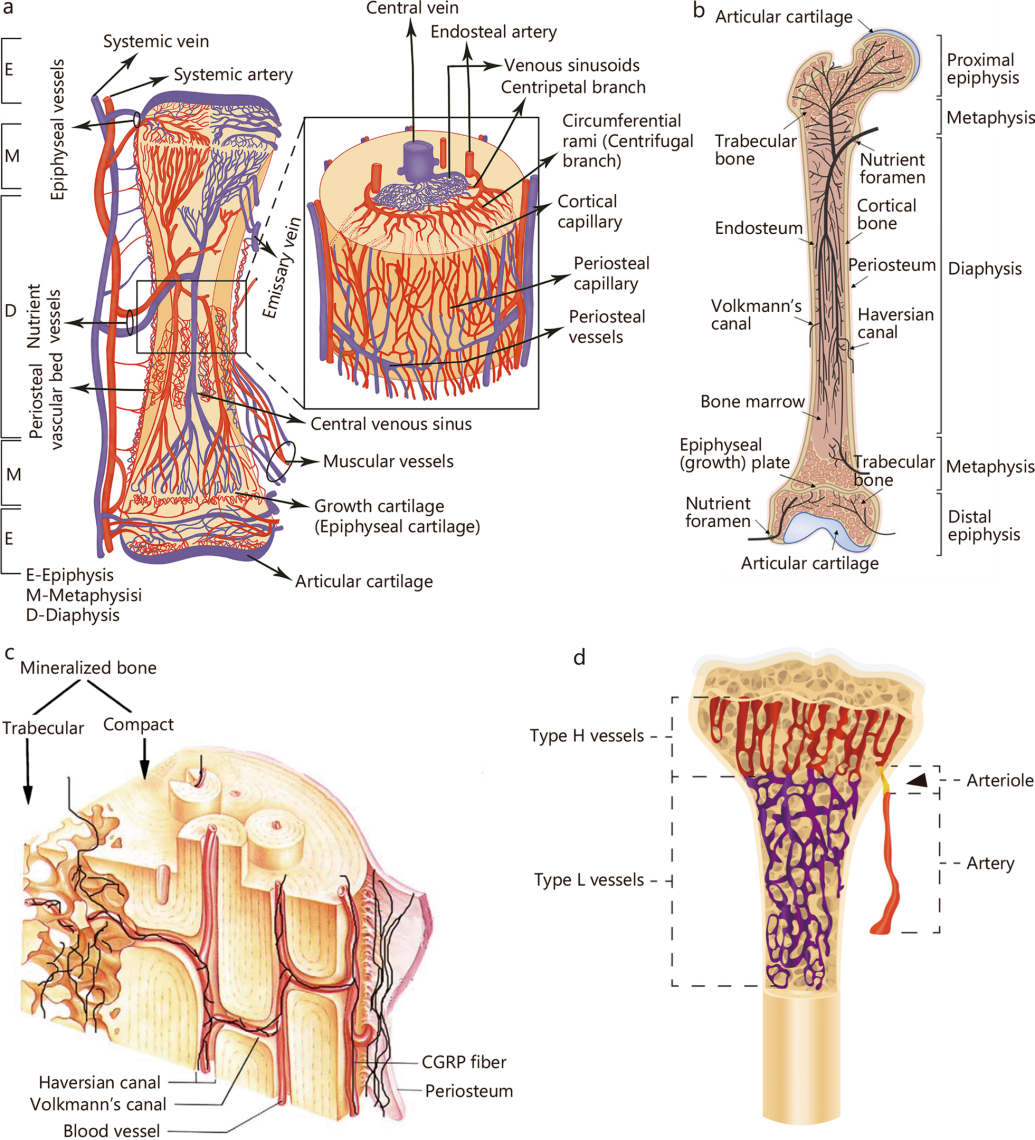
Distribution of the neurovascular system in bone. **a** The blood supply of a long bone. The marrow cavity contains a large central venous sinus, a dense network of medullary sinusoids, and longitudinal medullary arteries and their circumferential rami [33]. **b** A simplified schematic of the neuronal distribution in the mouse femur [34]. **c** A schematic illustrating the general pattern and course of the sensory nerve fibers and blood vessels in the periosteum and mineralized bone [11]. **d** A schematic of the morphology and distribution of type H and type L blood vessels. Arrowhead marks the entry of the arteriole through the cortical bone [35]. CGRP calcitonin gene-related peptide

The neurovascular system is extensively distributed throughout the skeleton, such as the periosteum, cortical bone, and bone marrow (Fig. 2) [11, 33–35]. However, the extent to which neurovascular coupling contributes to improvements in skeletal remodeling remains obscure. In this section, the role of neurovascular coupling in bone development, metabolism, and repair will be comprehensively discussed. By understanding these fundamental aspects, a clearer picture of how nerve-vessel interactions influence bone health may be formed, potentially guiding future research to address the existing knowledge gaps.

### Role of neurovascular coupling in bone development

Embryological anatomical studies show that neural tissue develops before bone tissue. Innervation first appears in the central part of the backbone, followed by the extension to the metaphysis. Subsequently, bone tissue forms canals surrounding neural pathways, thereby forming new bone [36]. Two bone formation processes including intramembranous ossification and endochondral ossification are present during embryogenesis [37].

Intramembranous ossification occurs during the development of flat bones including the skull, mandible, maxilla, and clavicle. Ossification begins with the condensation of mesenchymal stem cells (MSCs). These cells secrete growth factors such as vascular endothelial growth factor (VEGF)-A to promote the differentiation of MSCs into osteoprogenitors and osteoblasts. Ossification centers are eventually formed as blood vessels invade these centers to promote osteogenesis [38]. During endochondral ossification in long bones, chondrocytes become hypertrophic with a high expression level of VEGF. Blood vessels infiltrate the hypertrophic cartilage to induce the differentiation of osteoblasts and the formation of the bone marrow cavity [37]. The first capillary plexus continues to sprout longitudinally towards the ends of the bone. This causes the bone marrow cavity to expand from the center and form epiphyseal growth plates at both ends. Then ossified bone and bone marrow gradually replace the cartilage [39, 40]. In addition, the vascular system serves as an active secretor of many signaling molecules to regulate the growth, differentiation, patterning, homeostasis, and morphogenesis of developing tissues [41, 42]. Bone morphogenetic protein 2 (BMP-2) and BMP-4, released by vessel-forming cells, act on chondrocytes and osteoblasts [43]. In addition, factors including platelet-derived growth factor type BB (PDGF-BB), slit guidance ligand 3 (SLIT3), hypoxia-inducible factor 1-alpha (HIF-1α), and Notch signaling have been reported to be involved in osteogenesis-angiogenesis coupling [14].

Sensory and sympathetic nerve fibers are widely involved in both primary and secondary ossification processes. During postnatal development, interleukin (IL)-6 induced skeletal sympathetic cholinergic nerve fibers preserve the survival and function of osteocytes through the neurotrophic axis of the neurturin-GDNF-family receptor-α2 (NRTN-GFRα2) [44]. Meanwhile, perichondrial cells of the embryonic femur drive sensory nerve innervation of this bone element by secreting nerve growth factor (NGF). This cytokine promotes bone vascular invasion, progression of bone progenitor cell lineage, and eventually the formation of the primary ossification centers. These phenomena support a predominant role of skeletal innervation in bone development, which can affect the acquisition of peak bone mass.

Vascularization and innervation have many similarities in embryonic development and anatomy. Similar structures such as the growth cone in axons and tip cells in vessels orchestrated the chemotaxis of axons and blood vessels [45–47]. ECs secrete neurotropic factors such as artemin and neurotrophin 3 (NT3) to recruit and control innervation [48]. Sensory neurons and Schwann cells (SCs), in turn, secrete VEGF to regulate vessel growth in a spatiotemporally controlled manner [49].

### Role of neurovascular coupling in bone metabolism and health

The nervous system plays a regulatory role in bone metabolism and health [50]. Receptors for specific neuropeptides are present extensively in skeletal cells. The central nervous system (CNS) influences bone mass through humoral mechanisms. These mechanisms include the regulation of plasma calcium, and signals from the thyrotropic, hypothalamic-pituitary-corticotropic, somatotropic, and gonadotropic axes [18, 51]. Moreover, peripheral nerves communicate with the skeleton to regulate bone metabolism through nerve-resident cells, neuropeptides, axon guidance factors, and neurotrophins [31, 36, 52, 53]. These biological factors act directly on different target cells or organs to control bone metabolism and formation (Table 1) [54–64]. The sympathetic nervous system is particularly important in osteogenesis, as well as in bone metabolism and remodeling. Sympathetic nerves are regulated by leptin from the hypothalamus, which in turn regulates the outgoing sympathetic nerves to control bone mass [65]. Conversely, pathological conditions of the bone can affect nerve innervation [66–68]. For example, bone tumors can release neuropeptides that activate and sensitize the bone nociceptors and different areas of the nervous system, resulting in pain [69, 70].

**Table 1:** Effects of neuro-associated molecules on bone regeneration

Molecules		Receptors	Cell models	Animal models	Effect on bone	References
Neurotransmitters	NE	α-AR; β-AR	MC3T3-E1 cell line	α_1_B^−/−^ mice	Promote *Cebpd* gene expression and cell proliferation; Increase bone formation	Tanaka et al. [54]
	ACh	nAChRs; mAChRs	Mouse osteoblasts	α_2_nAChR^−/−^ mice	Regulate osteoblast proliferation; Enhance osteoclast apoptosis; Inhibit mineralized matrix resorption	Bajayo et al. [55]
Neuropeptides	CGRP	CRLR; RAMP1	Mouse osteoclast precursor cells; Mouse osteoblasts	A femoral fracture model in αCGRP^−/−^ mice	Promote osteogenic differentiation and adequate callus formation	Appelt et al. [56]
	SP	NK-1R	Rat MSCs	A femoral defect model in SD rats	Promote MSC recruitment and efficient osseointegration	Mu et al. [57]
	NPY	Y1R; Y2R	Mouse BMSCs	A femoral defect in NPY^−/−^ mice	Decrease cancellous bone volume; Inhibit bone formation rate	Baldock et al. [58]
Axon guidance factors	Sema3A	Neuropilin-1 & plexin-A	–	A calvarial defect model in SD rats	Inhibit RANKL expression; Increase callus and bone formation	Kenan et al. [59]
	Sema4D	Plexin-B1; plexin-B2	Mouse calvarial cells; Wild-type and Sema4d^−/−^ osteoclasts; Wild-type and Plxnb1^−/−^ osteoblasts	An OVX model in mice*,* Sema4d^−/−^ mice; and Plxnb1^−/−^ mice	Inhibit osteoblast differentiation by RhoA activation; Reduce bone formation	Negishi-Koga et al. [60]
	SLIT3	ROBO1	Mouse calvaria cells; Human BMSCs; MC3T3-E1 cell line; Mouse BMMs; RAW264.7 cells	An OVX model in mice, *Slit3*^*−/−*^ mice; and *Robo1*^*−/−*^ mice	Stimulate osteoblast migration and proliferation; Regulate bone remodeling	Kim et al. [61]
Neurotrophins	NGF	TrkA; p75NTR	–	A tibial fracture model in mice	Accelerate the conversion of cartilage to bone; Result in highly connected trabecular bone	Rivera et al. [62]
	BDNF	TrkB	MC3T3-E1 cell line; ST-2 mouse bone marrow stromal cells	An OVX model in SD rats	Promote osteoblast differentiation and mineralization; Reduce osteoclast formation in vivo	Park et al. [63]
	NT3	TrkC	MC3T3-E1 cell line	A proximal tibial defect in SD rats	Suppress chondrogenesis; Enhance osteogenesis and angiogenesis	Su et al. [64]

*ACh* acetylcholine, *AR* adrenergic receptor, *BMSCs* bone marrow mesenchymal stem cells, *BDNF* brain-derived neurotrophic factor, *BMMs* bone marrow-derived macrophages, *Cebpd* CCAAT/enhancer-binding protein δ, *CGRP* calcitonin gene-related peptide, *CRLR* calcitonin receptor-like receptor, *mAChRs* muscarinic acetylcholine receptors, *MSCs* mesenchymal stem cells, *nAChRs* nicotinic acetylcholine receptors, *NE* norepinephrine, *NPY* neuropeptide Y, *NGF* nerve growth factor, *NK-1R* neurokinin-1 receptor, *NT3* neurotrophin 3, *OVX* ovariectomized, *p75NTR* p75 neurotrophic factor receptor, *RAMP1* receptor activity-modifying protein 1, *RANKL* receptor activator of nuclear factor kappa-B ligand, *ROBO1* roundabout guidance receptor 1 *SD* Sprague–Dawley, *SP* substance P, *Sema* semaphorin, *Trk* tyrosine kinase, *Y1R* Y1-receptors, *Y2R* Y2-receptors

The vasculature plays a crucial role in the cross-talk between various cells within the bone tissue microenvironment. Type H vessels, found in the metaphysis and endosteum, are major regulators of bone metabolism. Any functional disruption in the Notch signaling pathway, which is a major regulatory pathway for type H vessels, reduces the abundance of ECs and type H vessels in the bone microenvironment [71, 72]. Factors such as PDGF-BB, SLIT2, SLIT3, HIF-1α, and VEGF have been shown to influence bone metabolism by affecting the formation of type H vessels [73–75]. HIF-1α acts as a cellular oxygen sensor and plays a key role in regulating bone homeostasis and angiogenesis [76]. HIF-1α has been reported to promote angiogenesis and osteogenic differentiation via the VEGF/Akt/mammalian target of rapamycin (mTOR) signaling pathway in adipose-derived stem cells [77]. Glucocorticoid-induced osteoporosis is treated by regulating the adenosine monophosphate-activated protein kinase/mTOR and HIF-1α/VEGF signaling pathways [78]. Matrix metalloproteinase (MMP)-2 inhibitor 1 induces osteogenesis differentiation of bone marrow mesenchymal stem cells (BMSCs) and promotes type H vessel angiogenesis to rescue osteoporosis through the HIF-1α signaling pathway [79]. MiR-26a-5p in extracellular vesicles derived from urine stem cells activates the HIF-1α/VEGF pathway by inhibiting histone deacetylase 4, promoting the differentiation of osteoblast progenitor cells and inhibiting osteoclast activity, and preventing diabetic osteoporosis [80]. SLIT2 and SLIT3, axon guidance molecules secreted by osteoclast lineages, function as local coupling factors that preserve bone balance and protect bone metabolism [61]. SLIT2 inhibits osteoclast differentiation and reduces the migration and fusion of preosteoclasts by suppressing recombinant cell division cycle protein 42 activity [81]. SLIT3 promotes bone formation and inhibits bone resorption through Robo receptors; it has strong therapeutic potential in metabolic bone diseases [82].

Evidence also suggests that neurovascular coupling has a significant impact  in bone metabolism. Calcitonin gene-related peptide (CGRP) is believed to promote bone formation partly due to its ability to dilate blood vessels and stimulate EC migration, promoting angiogenesis in bone remodeling [83]. In addition, substance P and neuropeptide Y secreted by sensory neurons can effectively activate ECs and promote angiogenesis. Sympathetic activation, however, may cause a decrease in type H vessels. MSCs can indirectly regulate EC angiogenesis through paracrine effects under conditions of sympathetic excitation [84]. These findings provide important evidence of the role of neurovascular coupling in bone metabolism. They offer a strong reference for understanding the treatment of bone-related diseases. Osteoporosis is a common metabolic bone disease in middle-aged and elderly populations. This condition is linked to Alzheimer’s disease, an age-related neurodegenerative disorder known for impairing memory and cognition. Alzheimer’s disease is associated with osteoporosis through an abnormal central serotonergic regulatory pathway [85, 86]. This pathway upregulates sympathetic nervous signaling, which in turn activates β-adrenergic receptor on bone cells, enhancing bone resorption [87–89]. Further elucidating these underlying mechanisms may provide new avenues for the prevention and treatment for bone diseases.

### Role of neurovascular coupling in bone repair

Bone repair is a complex, multi-step process that involves inflammation, neurovascular network reconstruction, rapid bone mineralization, and remodeling [90–94]. A bone defect, such as a fracture, creates a microenvironment of injury. The disruption of oxygen due to insufficient blood supply produces a hypoxic environment, which is an important physiological signal in the process of bone repair. This hypoxic environment regulates the production of key biological factors by osteoblasts; these factors affect EC proliferation, determine cellular differentiation, and induce ECs to secrete osteogenic growth factors [95, 96]. The accompanying inflammation and revascularization are the most crucial phases of bone repair [97]. Platelets first appear at the site of injury to form a blood clot or hematoma. High levels of VEGF-A within the hematoma promote vascular invasion. The hematoma also serves as a template for temporary vascular bone scab formation. This is followed by cartilage scab formation to stabilize the fracture. The cartilage scab contains osteoblasts that promote bone formation, chondrocytes that contribute to new cartilage formation, and fibroblasts. The cartilage scab then matures into a hard bone scab, which is finally remodeled into mature bone, forming new trabeculae and cortical bone. In a murine femoral fracture model, inhibition of VEGF signaling by Fms-related receptor tyrosine kinase 1 (Flt-1), delayed cartilage turnover, disrupted conversion of soft cartilaginous callus to a hard bony callus, and impaired fracture healing [98]. This unstable, hypoxic environment results in indirect bone healing that resembles endochondral osteogenesis [38, 99, 100].

**Fig. 3:**
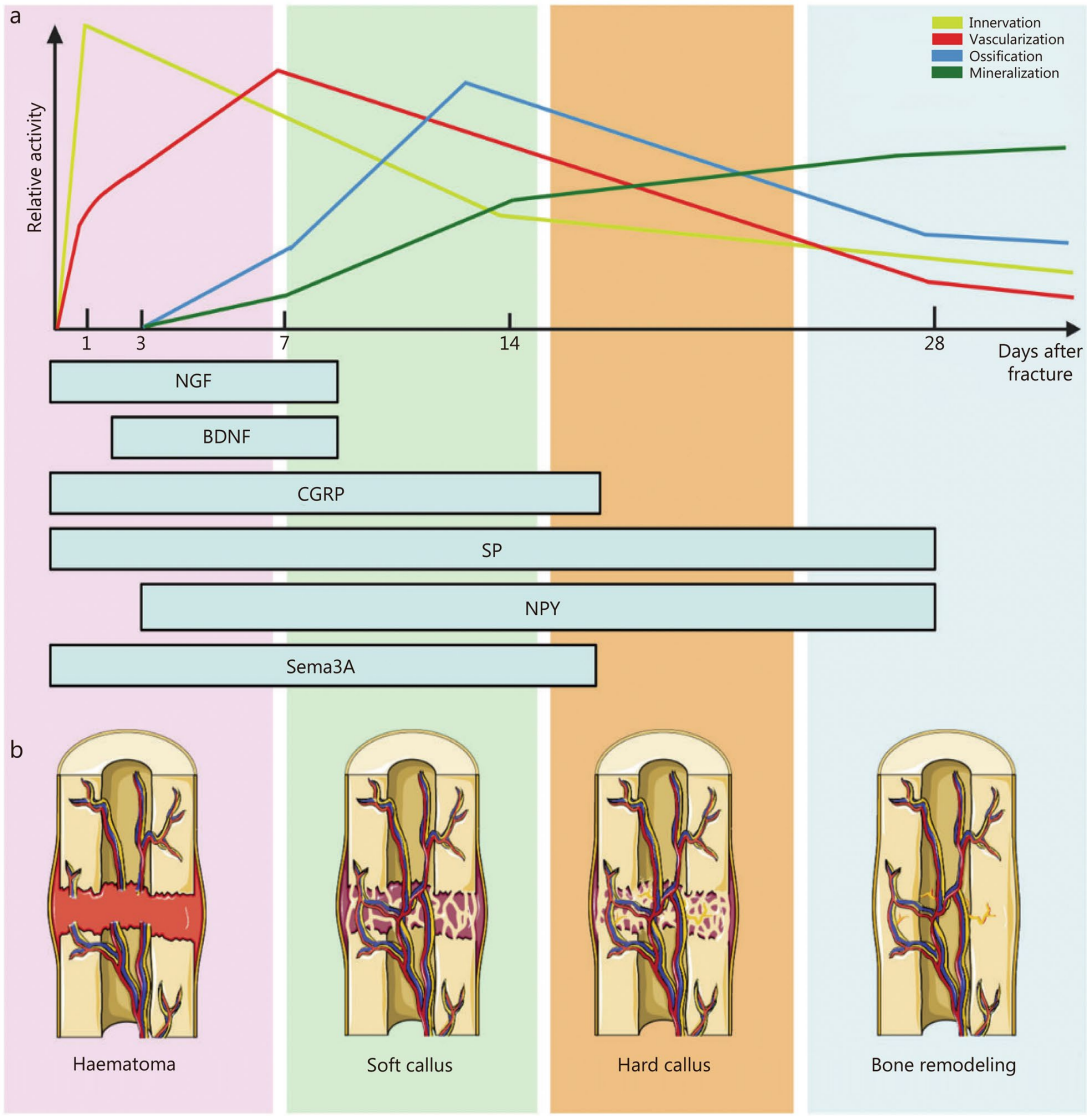
Expression of neuro-associated molecules and related events during bone healing. **a** The line chart displays the temporal sequence of events following a bone fracture. **b** Distinctive distribution of neuropeptides across the 4 phases of bone healing [17]. BDNF brain-derived neurotrophic factor, CGRP calcitonin gene-related peptide, NGF nerve growth nerve, NPY neuropeptide Y, Sema3A semaphorin 3A, SP substance P

Nerve fibers play a critical regulatory role not only in bone metabolism but also in fracture repair. It is reported that the healing process of fracture in patients with brain trauma was significantly accelerated [101]. Accelerating fracture healing after CNS injury may be related to factors such as the local release of growth factors in the brain [102–105]. The sensory nerves in peripheral nerves dominate bone repair by transmitting information about local conditions of bone fracture to the CNS and allowing the perception of pain signals. This can elicit an appropriate neuroendocrine response to modulate bone turnover locally at the fracture site [106]. In addition, sensory nerves are directly involved in osteogenesis through the secretion of neuropeptides, which stimulate the corresponding receptors on bone cells [107]. In the early bone repair phase, NGF was highly expressed in both macrophages and periosteal MSCs at the fracture site [108]. NGF not only stimulates the ingrowth of CGRP^+^ sensory fibers and tyrosine hydroxylase-positive (TH^+^) sympathetic fibers to the fracture callus from the periosteum and bone marrow, but also recruits MSCs to migrate toward bone defects and enhance bone formation via the p75 signaling pathway [109, 110]. Many other neuro-associated molecules are involved in different stages of fracture healing (Fig. 3) [17]. This substantiates that neurogenesis precedes vascular growth during bone repair. The interplay between nerves and blood vessels is intricate, with both entities collaborating to facilitate bone repair. Mg nail implantation has been shown to enhance the repair of critical-sized bone defects during distraction osteogenesis via CGRP/focal adhesion kinase (FAK)/VEGF signaling axis. This pathway may serve as a major signaling mechanism linking sensory nerve and ECs [111]. Research has demonstrated that CGRP has an equal effect on migration and tube formation compared to VEGF. Hence CGRP is a potent pro-angiogenic growth factor during bone healing [112].

In summary, neurovascular coupling plays a vital role in bone embryonic development, metabolism, and repair [72, 113]. Recently, there has been a growing body of research on how nerves and blood vessels affect bone regeneration [110, 114, 115]. However, studies that specifically address how neurovascular interactions influence bone-related conditions are still lacking. In addition, most research has focused on the effects of neuropeptides and cytokines. However, the clinical evidence of the application of neuropeptides and their receptor antagonists or agonists in conditions still has significant limitations [116]. Recently, cardiovascular research has introduced injectable and long-lasting CGRP analogues, which show antihypertensive effects, attenuate cardiac failure, and improve metabolic parameters in mice [117, 118]. Therefore, it is clinically important to investigate whether these novel CGRP analogues can not only be effective in treating metabolic and cardiac diseases, but also enhance innervated and vascularized bone regeneration, and whether their potential benefits outweigh the nociceptive effects known to be facilitated by CGRP. Investigating whether and how bone-organ axis regulates the intraosseous neurovascular system represents a promising area for future research. In the brain-bone axis, while the brain exerts dominant effects in bone metabolism, homeostasis, and disease progression, bones, in turn, signal to the brain to promote brain development and skeletal growth [12]. For example, psychological stress has been shown to induce changes in bone mass through activation of the hypothalamic–pituitary–adrenal axis, glucocorticoid signaling, and a blunted response to growth factors [119]. Estrogen and osteocalcin (OCN) are hormones involved in bone homeostasis. These hormones may also influence cognitive function by inhibiting neuronal apoptosis and activating the acute stress response through the inhibition of the peripheral nervous system [120–122].

## Materials for regenerating innervated and vascularized bone

The interdependence between biomaterials and biology is recently recognized [123]. In this relation, altering the composition, physical properties, and structural characteristics of materials can affect the basic physiological functions of cells, including cell migration, proliferation, and differentiation. In response, cells secrete extracellular matrix (ECM) components to remodel tissues, maintaining their mechanical properties and architecture [124]. This further illustrates the impact of material properties on biological functions at different levels (i.e., cells, tissues, organs, and the whole organism). Thus, understanding how materials influence functions of multiple cells is essential for designing biomaterials that can regenerate bone with proper nerve and blood supply [125]. Representative examples of biomaterials used for innervated and vascularized bone regeneration are summarized in Table 2 [110, 114, 126–144].

**Table 2:** Representative examples of biomaterials used for innervated and vascularized bone regeneration

Scaffold	Key active ingredient	Raw materials	Characteristics	Functions	References
Hydrogels	IKVAV	ELPs; PEG	Fine-tunable rheological property; Biodegradability; Biocompatibility	Improve the density of vessels; Promote neuron recruitment and neurite outgrowth	Dos Santos et al. [126]
	GeP@Cu	GelMA	Biodegradability; Conductivity; Sustained release of Cu^2+^	Improve antibacterial properties; Promote osteogenic differentiation of BMSCs; Accelerate innervation and angiogenesis	Xu et al. [127]
	BFP-1; QK; IK-19	Alginate; gelatin microspheres	Cytocompatibility; Biodegradability; Sustained release of BFP-1, QK, and IK-19	Promote neuronal axon extension and angiogenesis; Restore the structure and function of bone tissue	Li et al. [128]
	Mucin 1; VEGF; substance P	pNIPAM; collagen; HA	Biocompatibility; Glyco-modulatory biomaterial	Enhance tube and nerve formation; Accelerate innervation and angiogenesis during bone regeneration	Barik et al. [129]
	Mo_2_Ti_2_C3; MXene	Gelatin; acrylamide; chitosan; acetic acid	Conductivity; Biocompatibility	Promotes osteogenesis and neurogenesis in bone defects	Wang et al. [130]
	GPQGIWGQ	PEG	Cell-dictated degradation	Mimic native periosteum; Promote early-stage neurovascularization; Enhance biomechanical stability	Li et al. [131]
	rGO	GelMA	Biodegradability; Biocompatibility; Non-hemolytic	Improve mechanical properties; Promote myelination	Zhang et al. [132]
	SC-derived exosomes	GelMA	Sustained release of exosomes	Facilitate macrophage polarization toward M2 phenotype; Enhance osteogenesis of BMSCs by activating TGF-β1/SMAD2/3 signaling	Hao et al. [133]
	BP@Mg nanosheets	GelMA; PEGDA; β-TCP	Biodegradability; Biocompatibility; Higher swelling rate	Bilayer hydrogel to mimic bionic bone structure; Accelerate vascular infiltration and innervation during bone regeneration	Xu et al. [134]
	Akermanite	PDA; PLGA	Fine injectability; Biocompatibility; Sustained release	Activate sensory nerve cells to secrete CGRP, which upregulates osteogenic gene transcription via H3K27 demethylation; Promote osteogenic differentiation of BMSCs and bone regeneration	Gu et al. [135]
Fiber spinning	Cerium (III, IV)	Eggshell membrane; HPAA	Biocompatibility; No cytotoxicity; Suitable topographical property to mimic the periosteum	Facilitate local neuro-vascularization; Activate p38/MAPK or mTOR signaling pathway of macrophages	Wan et al. [136]
	SC-derived exosomes	PCL	Biocompatibility; Biodegradability; Target injured axons	Promote vesicle transport through the JNK/MAPK pathway; Promote blood vessel, nerve, and bone regeneration	Su et al. [137]
	siRNA@MMs	PCL	Biocompatibility	Inhibit the inflammatory cell infiltration; Promote the secretion of vascular and neurotrophic cytokines; Enhance the osteogenic differentiation of MSCs	Qiao et al. [138]
	WH@Nd	PCL	Biodegradability; Biocompatibility; Photothermal response; Sustained release of Ca^2+^, Mg^2+^, and PO^4−^	Imitate the double-layer structure of native periosteum; Simultaneous growth of nerves and blood vessels	Li et al. [114]
Hard scaffolds	BMP-2; NGF; VEGF	HA; silk	Biocompatibility; Excellent mechanical properties	Improve mechanical properties; Osteoconductivity; Early chemotactic migration of the HUVECs	Fitzpatrick et al. [139]
	WH	PCL	Piezoelectricity; Biodegradability; Biocompatibility; Sustained release of Mg^2+^	Promote angiogenesis and osteogenic differentiation of BMSCs; Depress osteoclast phenotype; Promote neurogenesis and angiogenesis	Wang et al. [140]
	Mg^2+^; Zn^2+^	α-TCP; gelatin microsphere; Zn-doped bioglass	Printability; Biocompatibility; Sustained release of Mg^2+^ and Zn^2+^	Promote osteogenic differentiation of BMSCs; Promote early vascularization and neurogenesis for bone regeneration	Xia et al. [141]
	NGF; BMP-2	Porcine dermis derived ECM	Sequential release of NGF (first 15 d) and BMP-2 (sustained release)	Enhance sensory nerve innervation; Accelerate innervation and angiogenesis during bone regeneration	Zhang et al. [110]
	NGF; MSC-exosomes	PLCL	Sustained release of exosomes; Biocompatibility	Induce myelinization and reinnervation; Activate the MAPK and PI3K/Akt signaling pathways	Lian et al. [142]
	daCO-decellularized matrix	PCL	Biocompatibility; Retain natural cellular matrix components; Remove immunogenic cellular DNA	Promote osteogenic differentiation and mineralization; Promote the formation of type H blood vessels	Wang et al. [143]
	Propranolol	GelMA microspheres; GelMA; HA	Sustained release of propranolol	Inhibit catecholamine release from the sympathetic nervous system; Promote recruitment and osteogenic differentiation of BMSCs; Promote bone regeneration	Su et al. [144]

*BMSCs* bone marrow mesenchymal stem cells, *BMP-2* Bone morphogenetic protein 2, *BP@Mg* magnesium-ion-modified black phosphorus, *CGRP* calcitonin gene-related peptide, *daCO* osteocytes derived from the mice with Wnt signaling activated, *ECM* extracellular matrix, *ELPs* elastin-like polypeptides, *GelMA* gelatin methacryloyl*, HA* hydroxyapatite, *HPAA* high-molecular-weight-polyacrylic acid, *HUVECs* human umbilical vein endothelial cells, *IK-19* Ac-KLTWQELYQLKYKGI-NH2, *MM* hybrid cell membrane, *MSCs* mesenchymal stem cells, *Nd* neodymium, *NGF* nerve growth factor, *pNIPAM* poly(N-isopropylacrylamide), *PEI* polyethylenimine, *PEGDA* polyethylene glycol diacrylate, *PDA* polydopamine, *PLCL* poly(L-lactic acid-ε-caprolactone), *PLGA* poly(lactic acid-co-glycolic acid), *QK* Ac-CSRARKQAASIKVAVSADR-NH2, *rGO* reduced graphene oxide, *TCP* tricalcium phosphate, *WH* whitlockite, *IKVAV* isoleucine-lysine-valine-alanine-valine, *PEG* poly (ethylene glycol), *BFP-1* bone-forming peptide-3, *VEGF* vascular endothelial growth factor, *SC* Schwann cell, *TGF-β1* transforming growth factor β1, *SMAD* small mothers against decapentaplegic homolog, *MAPK* mitogen-activated protein kinase, *mTOR* mammalian target of rapamycin, *PCL* polycaprolactone, *JNK* c-Jun N-terminal Kinase, *PI3K/Akt* phosphatidylinositol 3-kinase/ protein kinase B

### Material properties

Natural bone tissue is heterogeneous due to the varying density and distribution of bone tissues, blood vessels, nerves, and peripheral tissues, which makes it challenging to regenerate different tissues using the morphological characteristics of bioactive materials [145]. The physicochemical properties of materials, such as stiffness, surface topography, pore structures, conductivity, and piezoelectricity are crucial for tissue regeneration because these properties significantly influence cellular behaviors [146–149].

#### Stiffness

Stiffness is defined as the ration of stress to strain. It is considered a crucial biomechanical factor for bone tissue engineering [150]. Bone tissue comprises cortical and cancellous bone, with elastic moduli ranging from 10–25 GPa and 0.1–2.0 GPa, respectively [151]. An ideal scaffold material should match the stiffness of human bone to fulfill load-bearing function. Material stiffness could regulate cell behaviors such as cell adhesion, proliferation, migration, and differentiation, by making cells perceive the mechanical properties of ECM [150]. This process, known as mechanotransduction, involves cells converting extracellular physical sensations into intracellular biochemical signals [152]. Scientists found that osteogenic differentiation of MSCs was enhanced which were cultured on a stiff ECM (40 kPa) compared to a soft ECM (4.5 kPa) [153]. In another study, it was proved that the osteogenic differentiation of the MSCs occurred predominantly at 11–30 kPa, especially at 22 kPa in 3D hydrogel [154]. Mechanistically, the stiffer ECM regulates glutamine metabolism to contribute to osteogenesis, with Yes-associated protein playing an indispensable role in this process [152, 155]. Recent studies reported a significant role of the matrix stiffness in angiogenesis and neurotization. Exposure of human umbilical vein endothelial cells (HUVECs) to substrates of varying stiffness modulated the expression of major angiogenesis mediators and growth factors involved in bone repair and regeneration [156]. Collagen and hydroxyapatite (HA) mixtures in varying proportions were coated on decellularized cancellous bone to investigate the effect of stiffness on angiogenesis and bone regeneration. Compared to matrices with a stiffness of (13.00 ± 5.55) kPa, (13.87 ± 1.51) kPa and (37.70 ± 19.60) kPa showed higher expression of osteopontin, OCN, and increased aggregation of blood vessel-like ECs [157]. In addition, the cell spreading area and neurite length of differentiated neuron-like PC-12 cells significantly improved on 34.9 kPa gelatin methacryloyl (GelMA) substrates. This finding indicates that stiffer materials enhance cell adhesion and proliferation [158, 159]. It has been shown that osteogenic differentiation, vascular differentiation, and neural differentiation of stem cells differ when they are cultured on substrates with different stiffness [154, 160, 161]. When cultured on substrates with low, intermediate, or high stiffness, human MSCs differentiate into neurons, myoblasts, or osteoblasts, respectively. The stiffest substrate (25–40 kPa) demonstrated predominantly osteogenic differentiation [162]. Thus, determining the optimal stiffness of biomaterials is necessary for enhancing the growth and activity of different cell types to achieve neurovascularization in bone regeneration.

#### Surface topography

Careful assessment of surface topography is essential to enhance the regenerative capacity of bone. Similar to the stiffness, cells sense the material surface via adhesion receptors such as integrins. These molecules bind with intracellular functional proteins, triggering signal transduction and affecting further cellular responses to the implanted materials [150]. Surface topography plays a crucial role in regulating various cell behaviors including stem cell differentiation, myoblast migration, osteoblast maturation, and angiogenesis [163–166]. Both natural bone and blood vessel walls have some nanostructured surfaces composed of ECM proteins [124]. Surface modification is a well-known topographic feature for improved bioactivity, promotion of osteogenesis, enhancement of angiogenesis, moderation of pro-inflammatory responses, augmentation of anti-inflammatory responses, and reduction of osteoclast resorption activity [24, 167].

Methods for roughening smooth implants include sandblasting and acid etching, electrophoretic deposition, anodization, and hydrothermal treatment [168–173]. Micro-/nano-structured implants outperform smooth ones in both healthy and compromised animals [174–176]. A recent study investigated the effect of titanium nanotubes with different diameters (30 nm, 70 nm, or 110 nm) on osseointegration [177]. Titanium nanotubes with a 70 nm diameter significantly reduced early inflammation, promoted osteogenesis-angiogenesis coupling, and enhanced peri-implant osseointegration. These nanotubes also favored macrophage polarization towards an anti-inflammatory M2 phenotype through the FAK-phosphatidylinositide 3-kinase (PI3K)-mediated integrin α5/β3 pathway. Likewise, nano-structured surfaces of polymer substrates like polyurethane or poly(lactic-co-glycolic-acid) enhance the adhesion and growth of vascular-related cells such as ECs and vascular smooth muscle cells [178, 179]. Optimizing the topography and orientation of nerve tissue in biomaterials improves nerve tissue engineering outcomes by providing a suitable microenvironment for nerve cell growth and alignment [180].

In addition, a series of polyhedral bioceramic scaffolds fabricated through 3D printing and featured various spatial topologies [truncated tetrahedron, truncated octahedron, truncated cube, and cuboctahedron] were developed to enhance both nerves and blood vessels integration during bone regeneration. Notably, the truncated tetrahedron and truncated octahedron scaffolds exhibited the highest surface-to-volume ratio, providing increased surface area that supports greater cell adhesion and proliferation. This, in turn, facilitated both nerve regeneration and angiogenesis. Furthermore, these scaffolds demonstrated significantly higher compressive strength and featured tunable mechanical properties by design, ultimately promoting bone regeneration through activation of the PI3K/Akt signaling pathway [181]. These examples illustrate the importance of surface topography in designing biomaterials for effective tissue regeneration.

#### Pore structures

Pore structures with appropriate pore size and interconnected porosity are crucial for mimicking natural bone tissue [182]. Generally, large pore sizes favor blood vessel growth, while small pore sizes lead to poor contact between newly-formed tubes [183]. Connections between pores create anastomoses between capillaries, forming a functional vascular network. Polylactic acid scaffolds with a pore size less than 125 μm affect vessels penetration, while those with a diameter exceeding 250 μm promote vessel formation [184].

For Ti6Al4V scaffolds, the optimal pore size for angiogenesis is 550 μm [185]. A study using a 3D porous, biodegradable calcium phosphate scaffold found the optimal pore size for angiogenesis and bone formation is 540 μm. Scaffold porosity significantly influences arterial growth [186]. Scaffolds with a porosity of over 80% and a pore size of 400–600 µm are beneficial for bone regeneration [187–189]. Higher scaffold porosity increases the number of ECs, active osteoblasts, and bone mass. These findings indicate that angiogenesis and osteogenesis seem to depend on more porosity than pore size [190]. Although sufficient porosity is essential, inadequate connection and communication between pores may still result in poor vascularization [191]. The interconnection between pores plays a critical role in determining blood permeability and cell migration. These factors are vital for blood vessel formation within the scaffold [192]. 3D porous beta-tricalcium phosphate (β-TCP) scaffolds with identical pore sizes but varying interconnection sizes (100, 120, and 150 µm) were developed to investigate the effects of pore interconnection on angiogenesis. An in vivo study using a rabbit femoral condyle defect model demonstrated that a 150 µm interconnection size significantly improved vascularization by activating the PI3K/Akt signaling pathway [193].

The optimal microstructural parameters of conduit designs seem 50–70% porosity, 5–30 μm pore size, and approximately 600 μm wall thickness for nerve regeneration [194]. Interconnected macropores are crucial for nutrient transport, cellular infiltration, and bone ingrowth, while micropores around 10 μm promote protein adsorption, cellular adhesion, and ion exchange. More research is needed to determine the optimal pore sizes for bone repair materials to support neurovascularization effectively [195, 196].

#### Conductivity

Conductive biomaterials have been studied extensively since their introduction in 1994 [197]. They possess high electrical conductivity and electrochemical properties, enhancing cellular signaling in damaged tissues [198–201]. Research has shown that electrical stimulation aids in the repair and regeneration of damaged tissues including bone, blood vessel, nerve, and ligaments [202]. Unlike external electrical stimulation, conductive biomaterials do not require external devices. This offers a new solution for promoting neurovascularization in bone regeneration, with significant clinical potential [203].

**Fig. 4:**
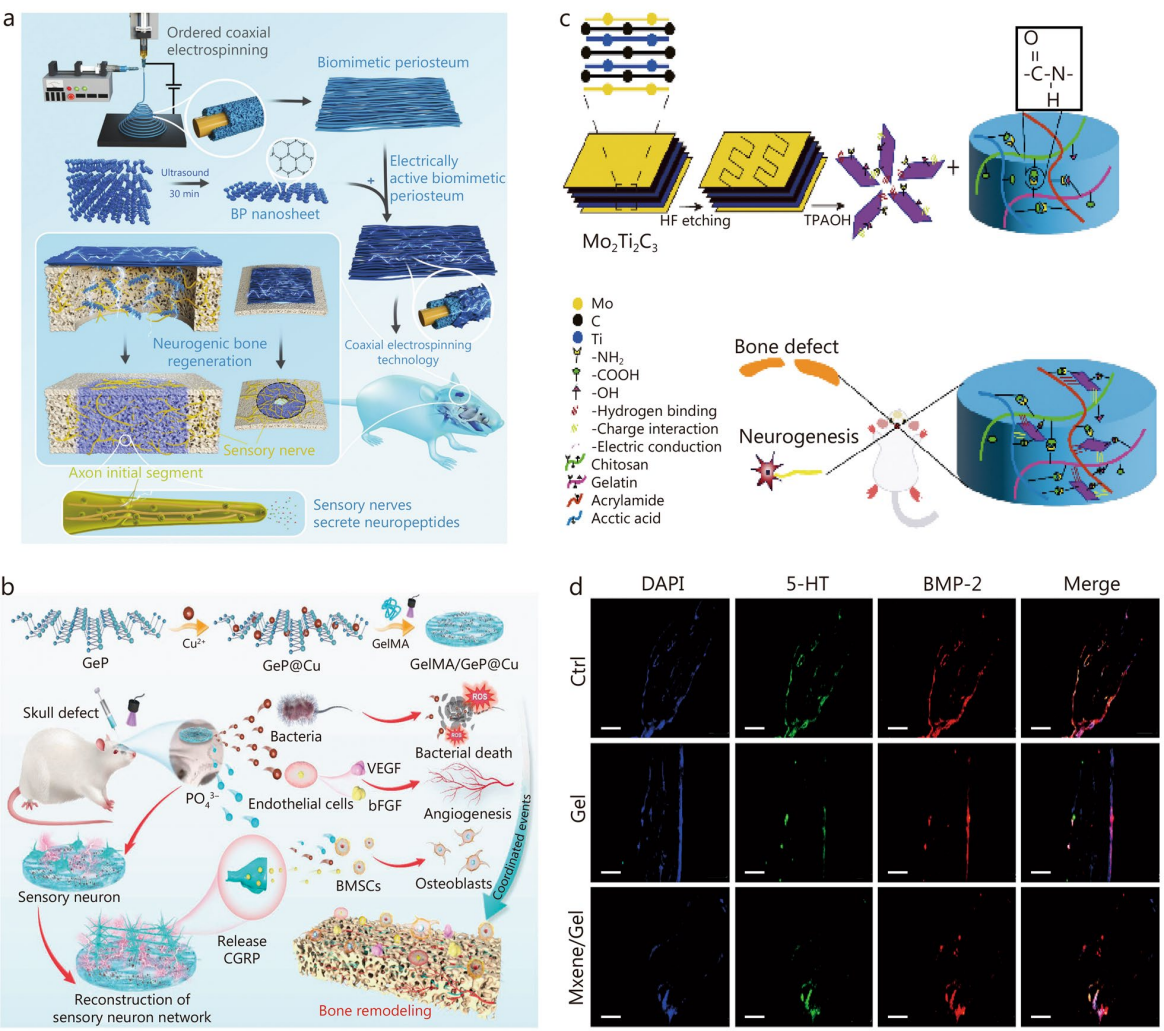
Conductive-based biomaterials promote innervated and vascularized bone regeneration.** a** Schematic of the (core)-polycaprolacton/(shell)–DNM biomimetic periosteum (PD)@black phosphorus promoting neurogenic bone regeneration [203]. **b** Schematic of the process used for the fabrication of the gelatin methacryloyl (GelMA)/GeP@Cu electroactive hydrogel and its multiple therapeutic actions supporting bone regeneration [127]. **c** Fabrication of Mo_2_Ti_2_C_3_ MXene hydrogel and its application in bone defects. **d** Immunofluorescence results showed MXene hydrogel increased the relative intensity of 5-hydroxytryptamine (5-HT) and bone morphogenetic protein 2 (BMP-2) expression at 8 weeks [130]. Scar bar = 100 μm. bFGF basic fibroblast growth factor, BMSCs bone marrow mesenchymal stem cells, CGRP calcitonin gene-related peptide, HF hydrofluoric acid, ROS reactive oxygen species, TPAOH tetrapropyl ammonium hydroxide, VEGF vascular endothelial growth factor

Black phosphorus is an isomer and the most stable form of phosphorus at room temperature and pressure. Black phosphorus nanosheets have superior conductivity, making them ideal for electrical stimulation in bone and nerve regeneration [134]. A core–shell electroactive membrane, developed using coaxial electrospinning technology, was designed to mimic natural bone membranes. These nanosheets were introduced onto the biomimetic periosteum via electrostatic interaction to enhance conductivity. This electrically-active periosteum enhances axon growth, promotes neurotransmitter secretion, and induces neurogenic osteogenesis (Fig. 4a) [204]. However, black phosphorus degrades in oxygen and water, which limits its biomedical applications [205]. Modification strategies such as polymer coating, surface chemical modification, and cell membrane embedding have been used to address this issue [206, 207]. In addition, copper ion-modified germanium phosphide nanosheets have been used to prepare a conductive GelMA hydrogel. This hydrogel demonstrated excellent innervation and vascularization during bone regeneration, with the added benefit of bacterial clearance during wound healing (Fig. 4b) [127].

A biocompatible and electrically conductive Mo_2_Ti_2_C_3_ MXene hydrogel was prepared using Mo–Ti to replace the Ti–Ti structure (Fig. 4c). The hydrogel upregulated the expression of the sensory neuron marker 5-hydroxytryptamine and the osteogenic factor BMP-2 (Fig. 4d) [130]. These results confirmed that the hydrogel promotes neurogenesis and bone regeneration in vivo. Despite these achievements, there is a lack of systematic analysis and evaluation of the long-term biosafety of MXene nanomaterials and their degradation products. Future research should focus on understanding the immunogenicity, pharmacokinetics, biological distribution, and long-term toxicity of these materials in animal models [208]. The development of conductive biomaterials for tissue engineering is still in early stages, facing significant scientific and technological challenges. Future efforts should improve features such as biodegradability, biocompatibility, stability, and compatibility to bridge the gap between laboratory findings and clinical applications.

#### Piezoelectricity

Unlike conductive materials, piezoelectric materials can immediately generate electrical signals in response to mechanical stress. This helps eliminate the risk of tissue damage from external electrical stimulation [209–211]. Because natural bone itself exhibits piezoelectric properties, different piezoelectric polymers such as polyvinylidene fluoride, polyhydroxyalkanoates, and barium titanate have been used for bone repair. These materials are selected because of their flexibility, ease of synthesis, and ability to offer an electrical stimulus [212–215]. However, their limited degradability and lack of bioactive components are insufficient for promoting neurovascularized bone regeneration [216, 217]. Whitlockite (WH), an inorganic nanoparticle with a composition similar to HA, releases Ca^2+^, Mg^2+^, and PO_4_^3−^ ions during degradation, and the released Mg^2+^ ions are known to support nerve and blood vessel formation [218]. Moreover, WH exhibits good piezoelectric properties upon sintering [219]. For example, a 3D-printed polycaprolactone/piezoelectric WH (PWH) composite scaffold was developed to mimic natural bone functionality. This scaffold enhances angiogenesis, promotes neuronal differentiation, suppresses osteoclast activity, and ultimately improves osteogenesis. The synergistic effect of bioactive ion release and piezoelectricity in this biodegradable PWH scaffold supports innervated and vascularized bone regeneration [140].

Furthermore, PWH has been utilized to reestablish the piezoelectric properties of natural periosteum, an important factor in bone healing. A double-network hydrogel composed of chelated alginate, GelMA, and PWH was designed to emulate the viscoelasticity and piezoelectricity of natural periosteum. Combined with a bone-like substrate, this periosteum-inspired structure reproduces the heterogeneous architecture of native bone tissue. When subjected to low-intensity pulsed ultrasound stimulation, this bioinspired scaffold significantly enhances early vascularization and neurogenesis. Under dynamic physiological conditions, the double-layer scaffold can function as a self-powered electrical stimulator, accelerating bone regeneration. This provides a valuable reference for exploring the physical properties of materials optimized for neurovascularized bone regeneration [220].

### Acellular biological factors

Biomaterials alone are ineffective in promoting osteogenesis due to the lack of bioactive components. They fail to mimic the body’s dynamic responsiveness to bone defects. Seed cells are the foundation of tissue engineering and regenerative medicine. An ideal seed cell should have strong proliferation ability, strong environmental adaptability, and good tissue compatibility [17]. Various seed cells have been employed, including BMSCs, SCs, and ECs. In addition to the cell delivery strategy (Table 3) [132, 221–228], some acellular biological factors have been loaded into biomaterials to provide additional stimuli. The following subsections will highlight representative examples of innervated and/or vascularized bone regeneration.

**Table 3:** Cell delivery strategy for innervated and vascularized bone regeneration

Cell type	Scaffold materials	Main results	References
HBMSCs	Lap®-alginate-methylcellulose bioink	Promote osteogenic differentiation, blood vessel penetration; Increase bone mineral density over 8 weeks	Cidonio et al. [220]
BMSCs	Lap-GA	Promote CGRP-induced osteogenic differentiation; Enhance osteogenesis and angiogenesis	Li et al. [221]
BMSCs; RAOECs	GelMA; PLA-PEG-PLA	Increase osteogenic differentiation; Promote RAOEC proliferation, migration, branching, and lumen formation; Prompt eventual bone regeneration	Shen et al. [222]
OMSCs; ECs	n‐HA/PU	Promote osteogenesis and angiogenesis when OMSCs and ECs at an optimal ratio (0.5/1.5) in co‐culture treatment	Li et al. [223]
MC3T3-E1; HUVECs	GelMA; Alg; nano β-TCP	Promote osteogenic differentiation and angiogenesis	Zhang et al. [224]
BMSCs; SCs	CS nanowires; GelMA	Promote osteogenic differentiation; Enhance the neurogenic activity of SCs; Induce the ingrowth of nerve fibers into bone defects area	Zhang et al. [225]
HUVECs; RUVECs	LMS bioceramics; GelMA	Enhance the neural differentiation of PC-12 cells and SCs; Upregulate the blood vessel‐related protein expression in HUVECs; Promote bone regeneration in vivo	Qin et al. [226]
BMSCs; SCs	rGO/GelMA	Upregulate osteogenic genes and proteins; Promote SC myelination; Promote eventual angiogenesis and neuralized bone regeneration	Zhang et al. [132]
RBMSCs; RAECs	Bioceramics	Promote angiogenic and neurogenic differentiation; Accelerate new bone formation	Zhang et al. [227]

*Alg* sodium alginate, *AlgMA* alginate methacrylate, *BMSCs* bone marrow mesenchymal stem cells, *CS* calcium silicate, *CGRP* calcitonin gene-related peptide, *ECs* endothelial cells, *GA* GelMA&AlgMA, *GelMA* gelatin methacryloyl, *HBMSCs* human bone marrow stromal cells, *HUVECs* human umbilical vein endothelial cells, *LMS* Li–Mg–Si, *Lap* laponite, *n‐HA/PU* nano‐hydroxyapatite/polyurethane, *nano β-TCP* nano beta-tricalcium phosphate, *OMSCs* osteogenic‐induced differentiated MSCs, *PLA* polylactic acid, *PEG* polyethylene glycol, *RAOECs* rat aortic endothelial cells, *RUVECs* rat umbilical vein endothelial cells, *rGO* reduced graphene oxide, *RBMSCs* rabbit bone marrow-derived MSCs, *RAECs* rabbit aortic endothelial cells, *SCs* Schwann cells

#### Neurotrophins

NGF was the first discovered neurotrophin [229]. It is widely expressed in osteoblasts, osteoclasts, osteocytes, and osteochondrocytes. The neurotrophin family includes NGF, brain-derived neurotrophic factor, glial cell line-derived neurotrophic factor, neurotrophin (NT)3, and NT4/5 [18]. Neurotrophins and their receptors regulate osteoblastogenesis, osteoclastogenesis, chondrogenesis, and angiogenesis during bone formation and injury repair [230–232]. There are two receptors, tyrosine kinase receptor A (TrkA; high-affinity) and p75 neurotrophic factor receptor (p75NTR; low-affinity) [233–235]. Neurotrophins support many kinds of neural activities, including axonal growth, synaptic plasticity, cell differentiation, and myelination by activating distinct TrkA [236]. Additionally, the p75NTR promotes osteogenesis by stimulating the BMP/ small mothers against decapentaplegic homolog 1 (SMAD1) signaling pathway, and inhibits bone resorption by down-regulating receptor activator of nuclear factor-κB ligand (RANKL) expression [230]. By binding to these receptors, NGF initiates signaling cascades like MAPK and PI3K/Akt, which sensitize neurons and stimulate axon and dendrite growth [163, 230, 237, 238].

Moreover, NGF exhibits angiogenic properties [239–241]. Meanwhile, β-NGF was locally applied with collagen bone fillers for critical-sized bone defect repair in rats. The β-NGF promotes nerve growth and stimulates VEGF synthesis via TrkA and ERK2 pathways. Although the use of NGF in neurovascularized bone regeneration is promising, its vulnerability and short half-life require suitable delivery vehicles for controlled, sustained delivery [164, 165].

**Fig. 5:**
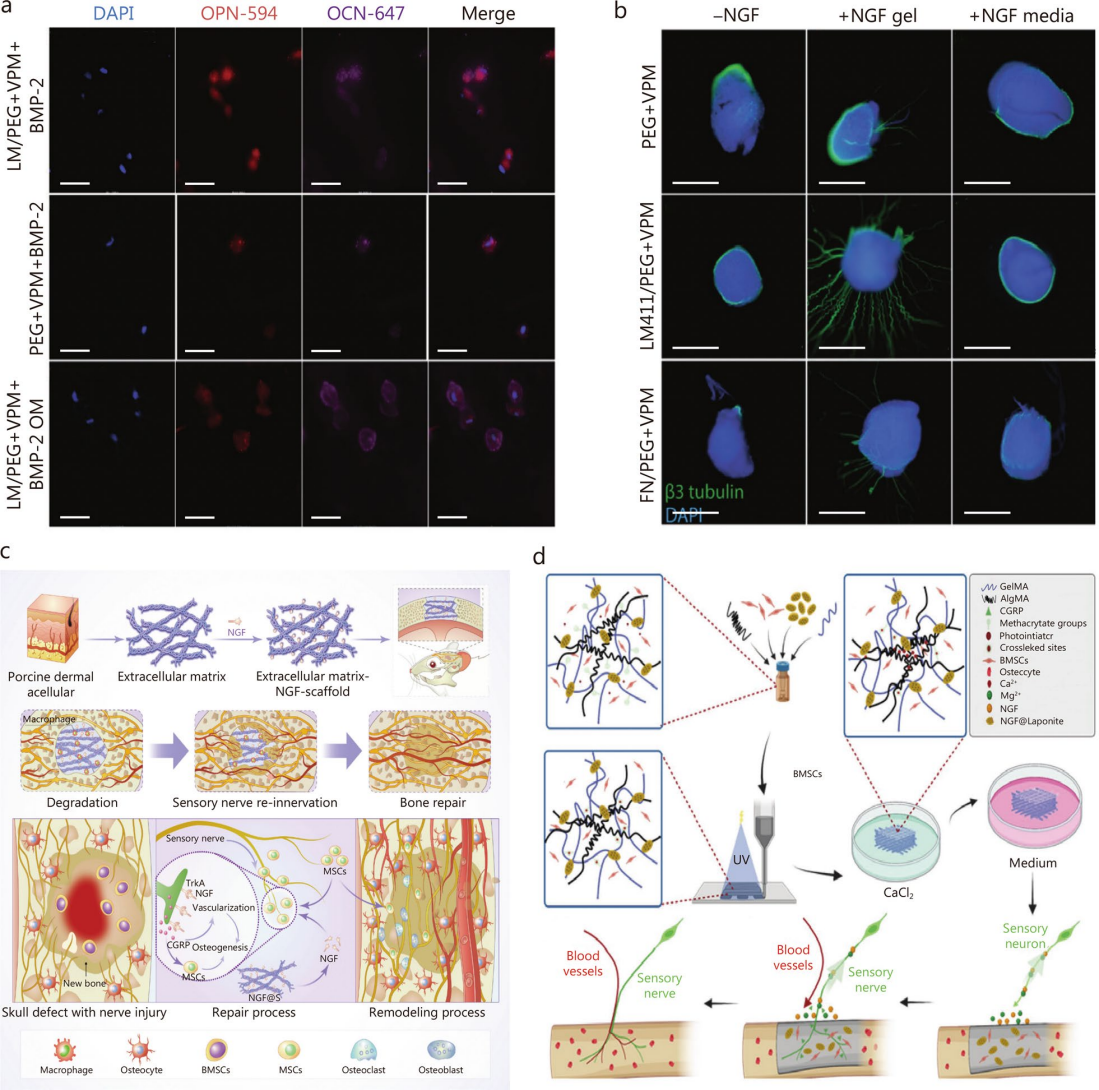
Strategies of delivering nerve growth factor (NGF) using biomaterials address burst release of NGF for innervated and vascularized bone regeneration.** a** Immunofluorescence results showed laminin (LM)332/polyethyleneglycol (PEG) effectively delivers bone morphogenetic protein 2 (BMP-2) and promotes the expression of late osteogenic markers osteopontin (OPN) and osteocalcin (OCN). **b** Immunofluorescence represented images of DRG cells in diverse culture conditions, which showed DRG cultured in LM411/PEG + GCRDVPMSMRGGDRCG peptide (VPM) hydrogels with 1 µg/ml of β-NGF showed the longest neurite outgrowth [165]. Scale bar = 500 µm. **c** The adsorption capacity of the acellular scaffold was leveraged to construct a sustained release system of NGF, which promoted sensory nerves reinnervation and bone repair [110]. **d** The schematic diagram showed the preparation of bioprinted constructs, which promote bone regeneration through sensory nerves and blood vessels regeneration [221]. AlgMA alginate methacrylate, BMP-2 bone morphogenetic protein 2, BMSCs bone marrow mesenchymal stem cells, CGRP calcitonin gene-related peptide, DAPI 4',6-Diamidino-2-phenylindole, DRG dorsal root ganglia, FN fibronectin, GelMA gelatin methacryloyl, MSCs mesenchymal stem cells, OM osteogenic media, TrkA tyrosine kinase receptor A, UV ultraviolet

Laminins (LMs) are important structural proteins in ECM, containing domains with affinity for growth factors. Two LM isoforms, LM332 and LM411, bind to BMP-2 and β-NGF, respectively. LM/polyethyleneglycol (PEG)-based hydrogels enhance BMP-2 and β-NGF bioactivity and stability, promoting bone and nerve regeneration (Fig. 5a, b) [166]. When NGF binds to porcine dermis-derived ECM nanofibrous scaffolds, it promotes bone defect healing by repairing damaged sensory nerves. NGF activates the TrkA receptor and stimulates CGRP secretion to promote angiogenesis (Fig. 5c) [110]. Laponite, a synthetic 2D silicate, fixes NGF in biomaterials by electrostatic adsorption [242]. Laponite loaded with NGF and BMSCs in a hybrid hydrogel regulates nervous system function, vascularization, and ossification to form functional bone tissue (Fig. 5d) [222]. Clinical trials suggest targeting NGF provides pain relief and improves physical function in osteoarthritic patients [180, 243]. This may be an effective target for pain treatment.

Brain-derived neurotrophic factor binds to TrkB and p75NTR, and is crucial for neuron survival and differentiation [230]. This neurotrophin also promotes angiogenesis and bone formation during human fracture healing via the TrkB [244]. Injecting NT3 promotes *BMP-2* and *VEGF* mRNA expression to enhance osteogenesis and angiogenesis [64]. Future efforts should explore the roles of other neurotrophins in bone and evaluate their clinical biosafety.

#### Peptides

Accelerating angiogenesis and innervation through peptides is a significant development in bone regeneration. Integrins, major cell-surface adhesion receptors, play key roles in cell spreading and proliferation [245]. Several bioactive peptides bind integrins to improve wound healing. Peptides such as Cys-Ala-Gly, Arg-Gly-Asp-Val (REDV), and Ser-Val-Val-Tyr-Gly-Leu-Arg have high affinity with ECs [246–248]. The REDV peptide, known for selective adsorption and proliferation of ECs, is used in surface modification of biomaterials for bone regeneration [249, 250]. A scaffold coated with REDV promotes early intrabony vascularization by binding to α_4_β_1_ integrin to attract ECs [251].

Bioactive epitopes in LM include Tyr-Lle-Gly-Ser-Arg (YIGSR) and Ile-Lys-Val-Ala-Val (IKVAV) [252, 253]. The YIGSR peptide guides EC migration, whereas IKVAV enhances EC mobilization, and capillary branching [254, 255]. Grafted into elastin-like polypeptides and branched PEG, these peptides form a composite matrix that, when implanted, lead to osteoid tissues with bone cells, vascular networks, and neuronal structures. However, the limited microporosity of PEG-based hydrogels prevents adequate cell infiltration after in vivo implantation. To address this issue, an all-natural polymer matrix was developed that incorporates ELPM80-alkene, biomimetic peptides containing IKVAV and YIGSR adhesion sequences, the lipophilic Suppocire® NA 15 **(**SNA15), and HA particles. This formulation provides the components essential for promoting cell colonization, bone mineralization, and the integration of multiple tissues. The formulation was found to enhance innervated and vascularized bone regeneration [256].

The degradation of hydrogels is critical to coordinate tissue infiltration for successful bone healing. Specific degradation peptide sequences can alter hydrogel degradation, promoting target cell migration to injury sites [257]. MMP-cleavable peptides, recognized and degraded by MMPs, serve as triggers in degradable biomaterials. GPQGIWGQ, a commonly-used sequence, is susceptible to MMP-2, -9, and -14 [258]. MMP-2 and -9 degrade ECM, enabling axonal outgrowth and neural cell pathfinding. Conversely, MMP-14 allows ECs to cleave ECM for lumen formation [259–261]. A hydrogel formed by polyethylene glycol and GPQGIWGQ was developed as a biomimetic periosteum for repairing bone defects [131]. This biomaterial supported EC migration in vitro, increased neurovascularization and enhanced bone regeneration in vivo. However, peptide degradation rates vary and are susceptible to MMP subtypes [262]. Given the complex and dynamic intercellular microenvironment, more than one MMP may be present at injury sites. Thus, MMP-cleavable peptide-based hydrogels require further investigation to be clinically applicable for bone regeneration.

#### RNAs

Gene therapy offers an alternative to protein therapy, and the first clinical trials began in the early 1990s [263]. RNAs-based therapeutics, such as mRNAs, microRNAs (miRNAs), small interfering RNA (siRNAs), and long noncoding RNAs, provide new approaches for treating bone diseases [264, 265]. These therapeutics can flexibly express proteins locally and intracellularly. Protein production may be maintained in-situ longer, reducing the need for higher therapeutic protein levels [266]. Delivery of RNAs shows potential to modulate neurobiological and angiogenesis processes, offering new opportunities for bone tissue engineering [267, 268].

##### miRNA

miRNAs are endogenous small noncoding RNAs (approximately 22 nucleotides) post-transcriptionally regulating gene expression [269]. Many miRNAs modulate neurobiological processes, including axonal outgrowth, synaptogenesis, and neural plasticity [270–272]. Among them, miR-222 has the potential to improve innervation in bone tissue engineering [273–275]. Co-delivery of miR-222 and aspirin promoted neurogenesis and bone formation in vivo [276]. Several miRNAs, such as miRNA-126, miRNA-210, miRNA-21, and miRNA-675, promote angiogenesis during bone regeneration [277–280]. They target HIF-1α/VEGF signaling, to improve microcirculation status in the bone injury area to facilitate bone regeneration.

##### siRNA

siRNAs effectively silence genes post-transcriptionally in eukaryotic cells [281]. RNA interference is crucial in bone regeneration by modulating osteocyte proliferation, differentiation, and function [282]. Inhibiting soluble Flt-1 (sFlt-1) and p75NTR may enhance vascular and neural differentiation, aiding repair of bone defects. However, siRNA therapies face delivery barriers like membrane impermeability, nuclease degradation, and lysosomal degradation [283, 284]. Dual siRNA copolymers, loaded into hybrid cell membranes derived from anti-inflammatory macrophages and osteogenic-induced MSCs, have been employed to address these delivery barriers [138]. This method creates a better bone defect microenvironment by improving angiogenesis, neurogenesis, and inflammatory regulation.

Despite progress, miRNAs and siRNAs applications in bone regeneration are limited to early preclinical trials. RNAs-based therapeutics face challenges in controlling entry routes, gene targeting, and determining optimal dosages.

#### Inorganic ions

Inorganic ions play an important role in bone repair and regeneration by regulating cellular behaviors and improving the bone microenvironment [285–288]. For instance, zinc ion has strong antibacterial activity, which can promote the healing of infected bone defects [289]. Metal ions also promote nerve and blood vessel regeneration in bone [134, 290]. Therefore, integrating bone scaffolds with inorganic ions is a promising therapeutic approach for bone defect repair.

##### Magnesium

**Fig. 6:**
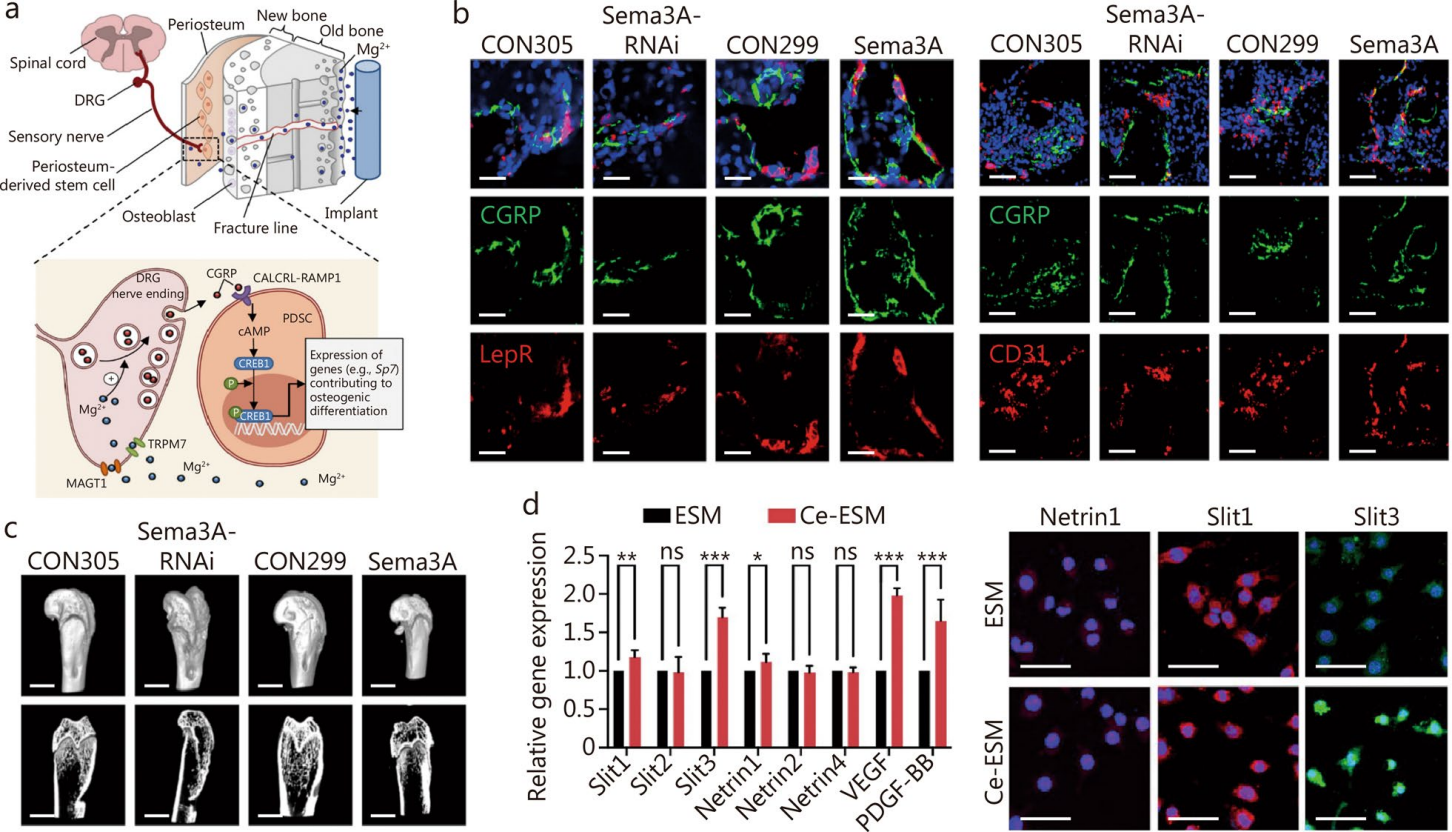
Inorganic ions-based biomaterials promote innervated and vascularized bone regeneration.** a** Schematic showing diffusion of implant-derived Mg^2+^ promotes osteogenic differentiation toward the periosteum that is innervated by sensory neurons [292]. **b** Immunofluorescence staining of overexpressing semaphorin 3A (Sema3A) in sensory nerves showed a large number of Leptin receptor (LepR)^+^ cell, Calcitonin gene-related peptide (CGRP)^+^ nerve fibers, and CD31^+^ vessels. Scale bar = 100 μm. **c** 3D-reconstructed superficial and interior images of femoral condyle defects showed overexpressing Sema3A in sensory nerves could accelerate bone regeneration [304]. Scale bar = 100 μm.** d** Ce-eggshell membrane (ESM) enhanced gene expressions of vascular endothelial growth factor (VEGF), platelet-derived growth factor-BB (PDGF-BB) and immunofluorescence images demonstrated a significant upregulation of SLIT3 in macrophages after Ce-ESM simulation [136]. *P < 0.05; **P < 0.01; ***P < 0.001, ns non-significant. Scale bar = 50 μm. cAMP cyclic adenosine monophosphate, CALCRL calcitonin receptor-like receptor, CGRP calcitonin gene-related peptide, CREB1 cAMP-responsive element binding protein 1, DRG dorsal root ganglion, MAGT1 magnesium induces magnesium transporter 1, PDSC periosteum-derived stem cell, RAMP1 receptor activity-modifying protein 1, SLIT slit guidance ligand, TRPM7 transient receptor potential cation channel subfamily M member 7, VEGF vascular endothelial growth factor

Magnesium is essential for bone health, with approximately 60% stored in the bone matrix [291]. Magnesium ions increase the proliferation and function of stem cells, promoting peripheral nerve repair [292]. Implant-derived Mg^2+^ enters sensory neuron dorsal root ganglia and promotes CGRP-vesicle accumulation and exocytosis, which enhances osteogenic gene expression in periosteum-derived stem cells (Fig. 6a) [293].

However, adding Mg^2+^ to biomaterials presents some challenges. Magnesium ions show a stage-dependent therapeutic effect. In the late stage of osteogenesis, Mg^2+^ upregulates Matrix Gla protein, a mineral-binding  ECM protein, which, in turn, inhibits HA crystal formation. Moreover, Mg^2+^ can replace Ca^2+^ in HA, inhibiting mineralization and osteogenesis. Controlling Mg^2+^ release is crucial in bone tissue engineering [294–296]. A 3D-printed dual-ion chronological release scaffold was designed. The early complete release of Mg^2+^ can effectively enhance neuro-vascularization without the potential inhibition on late osteogenesis, while long-term release of Zn^2+^ is responsible for promoting new bone formation [297–300].

##### Silicon

Silicon is the major trace element in the human body and is known for its positive effects on osteoblasts, osteoclasts, and ECs [301–303]. For instance, silicon-doped HA coatings on titanium implants promoted HUVEC migration and tube formation. These silicon-doped coatings also enhanced the expression of osteogenic markers in MC3T3-E1 cells, compared to HA-coated implants [304]. However, its impact on nerve regeneration is unclear. A silicified collagen scaffold has been shown to induce semaphorin 3A secreted by sensory nerves. In turn, semaphorin 3A stimulates neurovascularization in bone regeneration (Fig. 6b, c) [305].

Inorganic materials such as calcium silicate nanowires, nanoclays, and lithium-magnesium-silicon bioceramics have been used experimentally to assist bone regeneration. These biomaterials release biologically-active ions like Mg^2+^, Si^4+^, and Li^+^ to promote innervated and vascularized bone regeneration [222, 226]. The combined effect of multiple inorganic ions in biomaterials may exceed the role of a single ion.

##### Cerium

Cerium is recognized for its anti-inflammatory properties and its ability to enhance angiogenesis, neuroprotection, and bone repair [306]. Cerium can switch its oxidation state between cerium III and cerium IV. This property endows cerium oxide with attractive bio-catalytic and immunomodulatory properties for regulating the bone microenvironment [307]. Cerium (III, IV) oxide-mineralized ESMs (Ce-ESMs) was prepared through biomimetic mineralization to simulate natural  periosteum. The Ce-ESMs demonstrate superior mechanical properties and immunomodulatory capabilities. Under Ce-ESM stimulation, macrophages transform into tartrate-resistant acid phosphatase (TRAP)^+^ pre-osteoclasts. These active pre-osteoclasts secret VEGF, PDGF-BB, and SLIT3 to orchestrate bone regeneration and neurovascularization (Fig. 6d) [136].

##### Calcium

Calcium ions play an important structural role in bones, blood vessels, and nerves. Approximately 99% of the body’s calcium is found in the bones and teeth. The calcium is stored as carbonated apatite, which is the primary mineral phase of bone. The apatite crystallites provide strength to the skeletal system and serve as a metabolic reservoir for cellular fluids [308, 309]. In addition, calcium stimulates angiogenesis by promoting the proliferation of ECs, and upregulates the expression of VEGF and basic fibroblast growth factor [297]. In neurons, calcium is essential for signal transmission across synapses. Synaptic transmission occurs when an action potential reaches a nerve terminal, causing Ca^2+^ channels to open. This results in a highly localized and transient increase in intracellular Ca^2+^ at the active zone. The Ca^2+^ trigger exocytosis of synaptic vesicle, release of neurotransmitters, and initiate synaptic transmission [310]. Although there is a lack of relevant research on calcium ions in the field of innervated and vascularized bone regeneration, this novel type of ionic material offers novel approaches and insights for innervated and vascularized bone regeneration.

##### Copper

Copper ions enhance angiogenesis by stimulating proliferation and migration of ECs. These ions also activate pro-angiogenic factors such as VEGF, basic fibroblast growth factor, TNF-α, and IL-1 [311–313]. Copper-containing biomaterials, including Cu-doped HA, Cu-doped TCP, Cu-doped bioglass, copper sulfate, and copper sulfide, are used extensively in bone repair [314–317]. For instance, copper nanoparticles have been incorporated into calcium phosphate cement (CPC) to create Cu-doped CPC. This Cu-doped CPC promotes osteogenic differentiation, proliferation of HUVECs, and in vitro tube formation. Hence, it has the potential to facilitate the repair of cancerous bone defects [318]. Similarly, copper ions show potential for nerve regeneration. As previously mentioned, GelMA/GeP@Cu exhibits excellent electrical conductivity and antibacterial properties. This bioactive material has been reported to upregulate the expression of neuronal class III β-tubulin 1 and microtubule-associated protein 2 in neuroectodermal stem cells. Hence, GelMA/GeP@Cu has the potential to stimulate neurite growth and neural differentiation [127]. However, it is important to note that excessive Cu^2+^ can have adverse effects, such as cytotoxicity and the induction of apoptosis. This is due to the production of reactive oxygen species via Fenton-like and Haber–Weiss reactions [319–322]. In a study, Cu^2+^ and human-exfoliated deciduous teeth-derived exosomes were combined with hyaluronic acid hydrogel to promote periodontal bone regeneration. A concentration of 5.0 μg/ml Cu^2+^ was found to significantly upregulate the mRNA expression of *OCN* and runt-related transcription factor 2 (*Runx2*) in human periodontal ligament stem cells. This effect was more pronounced compared to a concentration of 7.0 μg/ml. These findings suggest that 5.0 μg/ml of Cu^2+^ exhibits both osteoinductive properties and favorable cytocompatibility [323].

#### Exosomes

**Fig. 7:**
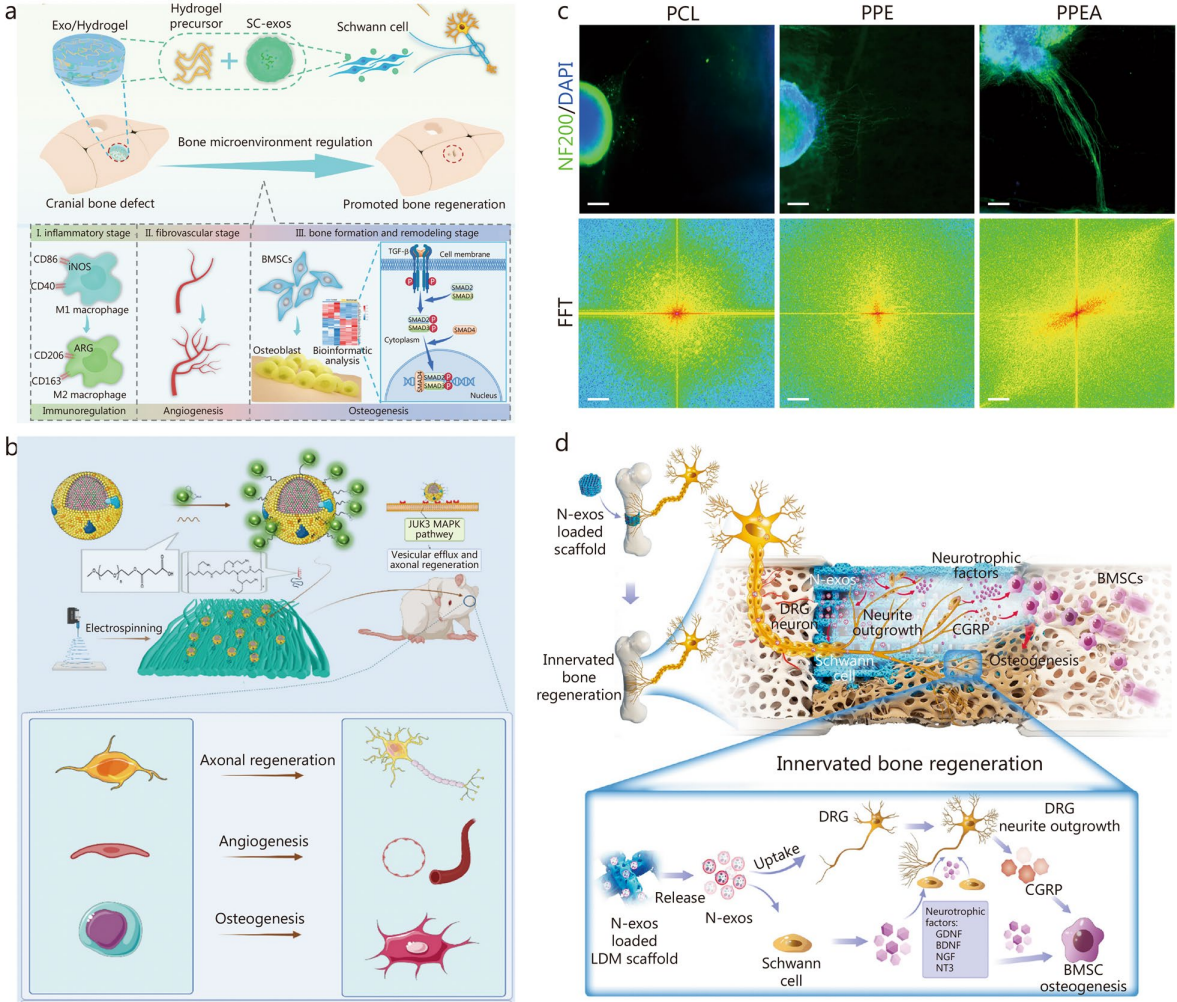
Natural and engineered exosome-based biomaterials promote innervated and vascularized bone regeneration.** a** At different stages of bone regeneration, Schwann cell-exosome (SC-exos)/hydrogel improves the osteogenic microenvironment and promotes neurovascularized bone regeneration [133]. **b** Schematic showing electrospun biomimetic periosteum loaded with aptamers engineered exosomes. These entities can target injured axons and regenerate blood vessels and bone. **c** Compared with the control group, aptamers engineered exosomes promoted dorsal root ganglion (DRG) axons growth and showed clear guidance [137]. **d** The N-exos-functionalized LDM-printed hierarchical porous scaffolds could promote the axonal growth and calcitonin gene-related peptide (CGRP) expression of sensory neurons and synergistically stimulate the osteogenic differentiation capacity of bone marrow mesenchymal stem cells (BMSCs) [142]. ARG arginase, BDNF brain-derived neurotrophic factor, Exos exosomes, FFT Fast Fourier Transform, GDNF glial-derived neurotrophic factor, iNOS inducible nitric oxide synthase, JUK3 c-Jun N-terminal kinase 3, LDM low temperature deposition modelling, MAPK mitogen-activated protein kinase, NF200 neurofilament 200, NGF nerve growth factor, NT3 neurotrophin-3, PPE PCL@PEI@exosome, PPEA PCL@PEI@exosome@aptamer, SMAD small mothers against decapentaplegic homolog, TGF-β transforming growth factor-β

Exosomes are considered as key mediators of cell-to-cell communication. They contain proteins, lipids, and nucleic acids (e.g*.*, mRNAs, small RNAs), which recipient cells absorb to exert functions [324]. Exosomes from different cell origins, such as BMSCs, umbilical cord MSCs, and endothelial progenitor cells, have been widely used in vascularized bone regeneration [277, 325, 326]. In the context of nerve regeneration, exosomes derived from SCs (SC-exos) have shown potential for treating peripheral nerve and spinal cord injuries. They can also enhance angiogenesis during nerve functional recovery [327, 328]. To ensure a sustainable and stable release, SC-exos were icorporated into GelMA. This assembly facilitated innervated and vascularized bone regeneration, with the potential for immune regulation (Fig. 7a) [133]. BMSCs and SC-exos were used as bioinks in GelMA and silk methacrylate hybrid hydrogels to mediate the SC-mediated nerve-bone crosstalk to promote osteogenesis [115]. These bio-printed constructs enhance neurovascularized bone regeneration by stimulating the nervous system.

However, nature exosomes still face challenges like large-scale production, stability, standard isolation, quality control, drug loading, and low targeting ability [133]. Engineered exosomes offer a solution by improving cell targeting and therapeutic effectiveness. Aptamer-engineered SC-exos were synthesized to phosphatidylserine on the cell surface to promote axonal fusion after axonal injury. The loaded SC-exos present on electrospun polycaprolactone membranes promoted axonal regeneration of dorsal root ganglia, tube formation by HUVECs, and healing of bone defects in vivo (Fig. 7b, c) [137]. In addition, NGF pre-stimulated MSC-containing porous scaffolds facilitated innervated bone regeneration in vivo (Fig. 7d) [142].

While these biomaterials have shown potential for promoting neurovascularized bone regeneration both in vitro and in vivo, further preclinical and clinical research is necessary to fully evaluate their osteogenic effects, application methods, and cost-effectiveness. Table 4 provides an overview of various material types and considerations for their selection [110, 114, 126, 127, 129–144]. Currently, research often lacks long-term biocompatibility assessments, focusing primarily on short-term effects on cell activity in vitro. Future studies should prioritize evaluating the metabolism of these materials in vivo, including their local and systemic toxicity to major organs. Another challenge lies in the choice of control materials in studies. Unmodified raw materials are frequently used as negative controls, while clinically established bone repair materials, such as Bio-Oss, BoneCeramic, and Puros, are not typically used as positive controls. Incorporating clinically used bone repair materials as positive controls will better validate the clinical potential of new materials.

**Table 4:** Overview of common material types for innervated and vascularized bone regeneration

Material type	Main advantages	Main disadvantages	Examples
Hydrogels	Good biocompatibility and stability; Cell-adhesion sites; Mimic extracellular matrix; Delivery platform of biological factors; Ease of modification	Low mechanical properties; Uncharted degradation rate in vivo	Gelatin [127, 130, 132–134] PEG [126, 131] Collagen [129] PLGA [135]
Fiber spinning	Good biocompatibility; High specific surface area; Mimic nature periosteum; Large-scale production; Good drug-loading performance	Low mechanical strength; Lack of bioactivity and osteoinduction	PCL [114, 137, 138] HPAA [136]
Hard scaffolds	Mechanical properties similar to natural bone; Mimic natural bone structures and properties; Tunable micro/nano topograph; Designable by CAD/CAM	Difficult to maintain long-term release of loaded biological factors; Slow biodegradability; Not applicable to irregular bone defects	HA [139, 144] PCL [140, 143] α-TCP [141] PLCL [142] Acellular matrix [110]

*PEG* polyethyleneglycol, *PLGA* poly(lactic acid-co-glycolic acid), *PCL* polycaprolactone, *HPAA* high-molecular-weight-polyacrylic acid, *HA* hydroxyapatite, *α-TCP* alpha-tricalcium phosphate, *PLCL* poly(L-lactic acid-ε-caprolactone), *CAD/CAM* computer-aided design/computer-aided manufacturing

## Preclinical studies and clinical trials

Despite the recent surge in bench-top and ex vivo studies, there remains a notable scarcity of preclinical and clinical research on the utilization of neurovascular bone grafting materials. In 2013, Kamburoǧlu et al. [329] reported that the prefabricated neuro-osseous flap-maintained bone metabolic activity and promoted neovascularization; they demonstrated the beneficial effect of the prefabricated flap on bone repair for the first time. Subsequent animal studies showed that vascularized fibula flaps and sural nerve grafting are effective to reconstruct long bone defects with extensive soft-tissue damages [330–332]. In addition, rib composite flaps with intercostal nerves and internal thoracic vessels have been shown to be promising for mandibular defect reconstruction [333]. Recent research indicates that innervated and vascularized iliac bone flaps offer advantages such as preserving lower lip sensation and effectively reducing bone resorption [334–336]. Neurovascularized allogeneic bone may be a potential candidate for bone tissue regeneration.

Biomaterials used for innervated and vascularized bone regeneration include hydrogels, fiber spinning, and hard scaffolds. The bone regenerative capacity of these biomaterials in vivo is influenced by multiple factors, including the type, microstructure, and pore characteristics of the biomaterials [337]. Other important factors are the animal species used, the defect size, and the implantation period, all of which can affect the behavior of biomaterials in vivo. Most applications of these biomaterials have been tested in small animal models such as mice, rats, and rabbits. The rat cranial defect model with a 5 mm in diameter is the most commonly used, due to the small size of rats, their ease of handling, and low housing requirements. However, rats have small, long bones with thin, weak cortices and do not exhibit Haversian-type cortex remodeling, unlike larger animals [338]. Moreover, rats experience ongoing growth or modeling due to their open growth plates. The use of fractures, osteotomy, and defect sites, as well as methods of internal and external fixation in rat models, does not closely match those used in clinical settings [339]. Rabbits are also frequently used because their mid-diaphyseal bone mineral density is similar to that of humans [340]. Nonetheless, the higher bone turnover rate and faster skeletal changes in rabbits make them an undesirable choice as a model for autogenous bone and marrow harvesting, processing, or transplantation. Furthermore, rabbits are notoriously sensitive to glucocorticoid stimulation, resulting in very oily marrow with physical properties that are distinctly different from human marrow [341, 342]. In summary, clear differences in bone microstructure and remodeling between small animal models and humans have been documented. The selection of animal models should match their similarity to the intended clinical application and mimic the underlying bone biology seen in human clinical settings. Therefore, future in vivo studies should consider using large animal models, such as bama minipigs, which have bone characteristics more closely aligned with human bone [343]. Pigs are considered the preferred animal model compared to sheep, despite their denser trabecular network. They are described as a highly representative model of human bone regeneration processes in terms of anatomical and morphological features, healing capacity, and remodeling, as well as bone mineral density and concentration [344, 345]. However, pigs are often overlooked due to the complexity of handling and the relatively small size of their tibia and femora [346].

Moreover, the presented study outcomes indicate that specific issues remain to be addressed before clinical translation. Medical-grade polycaprolactone-TCP scaffolds, as a second-generation scaffold, are currently in the Food and Drug Administration (FDA) preapproval stage. In a long-term (12-month) preclinical study, the scaffold failed to induce defect consolidation in a segmental tibial animal model (sheep). However, scaffolds combined with MSCs or recombinant human BMP-7 (rhBMP-7) showed significantly greater bone formation and superior strength compared to the autograft [347]. Additionally, various growth factors, such as BMP-2 and BMP-7, have been widely used in clinics and are approved by the FDA [348, 349]. The use of BMP promotes bone integration and improves the success rate of surgeries. However, BMP-2 has been reported as a dual-function cytokine that promotes ectopic bone formation through osteoinductive action and induces neuroinflammation [350]. Therefore, many challenges remain in translating preclinical studies to clinical trials.

Despite the encouraging results, all experiments in materials for regenerating innervated and vascularized bone (section "Materials for regenerating innervated and vascularized bone") were conducted in animal models, and none of these findings have yet been translated into human clinical trials. The unavailability of human clinical studies on neurovascular biomaterials for human bone regeneration highlights several challenges hindering clinical translation. Current studies primarily use small animals, such as mice, rats, rabbits, and guinea pigs. However, large animal models offer significant advantages due to their closer anatomical similarity to humans. The limited use of neurovascularization materials in preclinical studies, especially in large animals, poses a significant challenge. Previous study has shown significant heterogeneity in current surgical methods for inducing bone defects in rats, which reduces the reproducibility and comparability of preclinical studies [351]. There is still a need to develop standardized approaches for creating bone defect animal models to reliably verify the osteogenic performance of biomaterials. Further challenges include demonstrating and assessing dynamic material properties. The long-term biocompatibility and biodegradation rates of hard scaffolds, hydrogels, and spinners in vivo may vary due to their differing material properties. Therefore, it is necessary to design specific types of materials to match the corresponding healing rate of bone, depending on the characteristics of the defect sites. Moreover, there is a lack of comparative studies between neurovascular biomaterials and commercially available bone repair materials used in clinical settings. The benefits of incorporating novel material design features that result in only minor improvements in bone regeneration should be carefully weighed against the challenges of obtaining regulatory approval.

In action, ethical considerations, regulatory hurdles, and the high cost of developing and testing new treatments further complicate the progression from animal models to human trials. Many new applications fail to gain approval, even with positive clinical results, due to concerns from regulatory agencies. These concerns often include insufficient justification for clinical comparator selection, inappropriate endpoint design, and inadequate clinical data analysis methodology. For example, Opaxio’s European approval was denied despite a 42-day improvement in overall survival compared to the comparator. The developer of Opaxio later withdrew the application after European officials raised concerns over the clinical trial regimen in 2009 [352]. This example underscores the importance for both pharmaceutical companies and organizations developing biological materials to engage with regulators early and throughout the biomaterial development process. By seeking advice and addressing potential concerns proactively, companies can mitigate the risk of non-approval or delays.

To overcome these regulatory hurdles, the authors propose three solutions:

1. Enhanced preclinical testing: improving animal models to better simulate human bone biology, particularly by using large animal models like bama minipigs, would provide more reliable data before moving to human trials. This would address concerns about the limited applicability of small animal models.
2. Adaptive clinical trials: implementing adaptive trial designs allows for modifications based on interim results, enhancing both the safety and efficacy assessments while maintaining scientific rigor. This approach enables trials to respond dynamically to evolving data, potentially reducing the time to approval.
3. Collaborative approaches with regulators: establishing close collaboration with regulatory bodies, such as the FDA, from the early stages of development is crucial. Engaging in pre-submission meetings and incorporating feedback on trial design and data collection can prevent delays and streamline the approval process.

By addressing these challenges and incorporating these solutions, the clinical application of neurovascular bone repair materials may be advanced more efficiently.

Specific solutions also include identifying the specific requirements of targeted patients during the initial design stages. Due to the various causes of human bone defects, the clinical application of biological materials must take into account and compensate for patient-specific factors, as bone tissue can also be affected by the disease-related microenvironment. For example, in patients with bone defects related to malignancy, a biomaterials-only approach to bone tissue engineering may be preferable, as proliferation-stimulating biomolecules, such as growth factors, should not be introduced into former tumor sites [348]. To further enhance the neurovascularization capability of bone tissue affected by tumors, it is crucial to design biomaterials that incorporate additional elements beyond NGF, such as inorganic ions and exosomes, for optional use. Therefore, these materials should be subclassified based on the patient’s specific conditions or complications.

Involving specialist physicians with experiential knowledge of patient needs, clinical realities, and potential safety concerns is also crucial. In-depth communication between with biomaterial designers and clinicians is of significance to prevent cognitive differences as a result of common-sense understanding errors, differences in professional opinions, etc. Furthermore, different surgical methods for the same surgery, such as open suture or arthroscopic injection for implantation of cartilage implants, have different requirements for biomaterial structure and properties (e.g., viscoelasticity, strength, viscosity, injectability). Therefore, biomaterial researchers should establish a close relationship with clinicians, observe the diagnosis and treatment procedures in clinical practice, get to know the key requirements of clinical translation, and further seek solutions together [353].

In addition, it is essential to ensure that the designed materials are compatible with the evaluation standards of governmental regulatory agencies. For example, medical products in the field of tissue-engineered cartilage repair usually contain a combination of scaffold materials with cytokines and/or cells; different countries have different definitions of combined products. In China, combined products will be regulated as a single entity, whether it is a drug or a medical device based on certain products. In addition, biologics or cell- and tissue-based components are not separated from the drug class, unlike in Japan or the United States. In this process, the developers first determine the primary mode of action of the product, which determines whether the product’s properties are drug-led, bio-led, or medical-device-led. Products with different attributes have different requirements in production quality system, risk assessment, clinical evaluation, etc. Therefore, attention to which product type of neurovascularized bone regeneration material belongs to is crucial for governmental regulation, which profoundly affects the progress of product development and marketing [354].

## Conclusions and future perspectives

The mechanism and role of the neurovascular system in bone regeneration have garnered significant attention. Bones are covered by neural and vascular networks essential for the development, remodeling, and repair. Nerves promote blood vessel regeneration by secreting neurotransmitters and participate in various bone tissue activities. Blood vessels provide oxygen and nutrients to nerve fibers and bone tissue. Together, nerve fibers and blood vessels maintain the microenvironment for bone tissue regeneration, addressing clinical issues such as fractures and non-unions.

However, the crucial role of the neural networks in promoting bone regeneration is often overlooked in the design of biomaterials, which may result in delayed or compromised healing. The exact mechanism of how the neurovascular system regulates the bone defect microenvironment remains unclear. With the advent of neurovascularized bone regeneration materials, design strategies are diversifying, including neurotrophic factors, peptides, RNA, inorganic ions, and exosomes to promote neurovascularized bone tissue regeneration.

Despite advancements, most research has not yet reached the stage of clinical trials. Key factors include the lack of preclinical translational studies in large animals, the complexity of replicating the intricate neurovascular structures in engineered grafts, and the need for robust and reproducible methods to ensure safety and efficacy. Additionally, the impact of biomaterial properties on the intrabony neurovascular system is often overlooked. Effective neurovascularization in bone regeneration requires biomaterials that create a microenvironment meeting the functional needs of nerves, blood vessels, and bone tissue. However, regeneration of multiple tissues remains a bottleneck in bone tissue engineering, as bone regenerates better than nerves. Architectural design must address the contradictory requirements for regenerating the three tissues, such as stiffness, roughness, pore size and porosity, and conductivity.

Achieving clinical translation involves controlling the orderly growth of two tissues into the bone defect area to exert osteogenic effects. Future prospects for neurovascularized bone regeneration materials to facilitate clinical translation include:

1. As an emerging technology, organ-on-a-chip refers to a biomimetic micro-engineered system that mimics the microenvironment of native tissue and organs, based on a microfluidic chip that combines biology, materials science, and engineering. A microfluidic osteogenesis-on-a-chip device has been developed to simulate a 3D environment and fluid shear stresses [355]. Advances in microfluidic device fabrication techniques hold the potential to create more realistic and sophisticated pre-established peripheral vascular networks in bone grafts before implantation. However, challenges include the source of cells, scalability, standardization of manufacturing processes, and the limitations of chip size [356]. Moreover, no regulatory standards currently exist for organ-on-a-chip systems. It is essential for governmental regulatory agencies to develop ethical and regulatory guidelines to promote the advancement of this technology.
2. Osteo-organoids, which combine bioactive factors, scaffolds, and functional cells, have been used for various bone defect repairs [357]. An organoid-based strategy combined with 4D printing technology is expected to precisely fit the geometry of bone defects over time. Functional transformation during the post-printing stage may coordinate intrabony neurovascular system regeneration and facilitate dynamic bone remodeling. However, unlike single-material, non-cellular 3D-printed bone scaffolds that have been applied in clinical practice, bio-printed organoids are still in the early stages of development. Not only does the production technology need further refinement, but the storage and transport of organoids also present challenges. The ex vivo expansion of specific stem cells requires large-scale cell proliferation, which demands stringent expertise in both hardware and operational aspects [358]. Currently, organoid storage and transport rely primarily on long-term cryopreservation and short-term tissue preservation solutions, which are difficult to apply in clinical practice. Future in vivo studies should focus on developing more effective methods for organoid storage and transport.
3. Real-time monitoring of bone regeneration and resorption is currently achieved through techniques like fluorescence probes, prussian blue nanoparticles, superparamagnetic iron oxide nanoparticles, and sensors [359–362]. However, these technologies do not enable real-time monitoring of neurovascular regeneration in bone. Advanced imaging techniques, such as two-photon laser scanning microscopy combined with fluorescence technology, hold potential for real-time monitoring of blood vessels and nerves, though they require exposure of the monitoring site. Presently, sensing bandages and electroactive dressings can non-invasively monitor the healing process of skin defects by measuring indicators such as pH, resistance, temperature, and pressure [363–365]. Whether similar techniques can be applied to bone regeneration remains an area for future exploration. Advances in these technologies could facilitate more comprehensive efficacy evaluations of neurovascularized materials, leading to more accurate preclinical assessments of drugs and bone implant biomaterials.
4. Designing biomaterials that sequentially release bioactive factors is advantageous for simulating the physiological process of neurovascular regeneration during bone healing. Whether these bioactive factors could induce heterotopic ossification or cause biological complications remains an open question. Previous studies have reported a relationship between neurotrophins and pain during fracture healing [366–368]. Additionally, TrkA, the specific binding receptor for NGF, has been proven to be a potent carcinogenic driver when overexpressed [369]. Thus, it is crucial to carefully regulate the dosage and release rate of neurotrophins during the design of neurovascular biomaterials, particularly for patients with bone defects associated with malignancy [348]. Moreover, whether long-term accumulation of biomaterial degradation products could lead to complications also requires thorough investigation in preclinical studies.
5. Artificial intelligence (AI) and machine learning can automatically identify and extract key features from patients’ medical imaging data, such as the size, shape, and location of bone defects, providing precise guidance for scaffold design [370]. At the same time, AI systems can autonomously learn and optimize scaffold design parameters such as structural strength, biocompatibility, and biomechanical performance [371]. In addition, continuous ethical review, the establishment of data-sharing platforms, and the definition of data standards are crucial for advancing the role of AI in biomaterial research [372, 373]. To date, only a few AI models have been approved by the FDA in the field of orthopedic diseases [374–376]. Therefore, more robust datasets and AI models are needed to predict the formation of blood vessels and nerves, which would improve the design and application of biomaterials.

Advancement of these technologies has opened new vistas for the rational design of bioactive materials. In the future, scientists have to pay more attention to the crosstalk between blood vessels and nerves in bone, and their interaction with bone tissue. This is significant for guiding the design and clinical transformation of intraosseous biomaterials.

## Data Availability

Not applicable.

## Abbreviations

AI: Artificial intelligence
BMP-2: Bone morphogenetic protein 2
BMSCs: Bone marrow mesenchymal stem cells
Ce-ESMs: Cerium (III, IV) oxide-mineralized ESMs
CGRP: Calcitonin gene-related peptide
CNS: Central nervous system
CPC: Calcium phosphate cement
ECM: Extracellular matrix
ECs: Endothelial cells
FAK: Focal adhesion kinase
FDA: Food and Drug Administration
Flt-1: Fms-related receptor tyrosine kinase 1
GelMA: Gelatin methacryloyl
HA: Hydroxyapatite
HIF-1α: Hypoxia-inducible factor 1-alpha
HPAA: High-molecular-weight-polyacrylic acid
HUVECs: Human umbilical vein endothelial cells
IL: Interleukin
LMs: Laminins
miRNAs: MicroRNAs
MMP: Matrix metalloproteinase
MSCs: Mesenchymal stem cells
mTOR: Mammalian target of rapamycin
nano β-TCP: Nano beta-tricalcium phosphate
NGF: Nerve growth factor
NT3: Neurotrophin-3
NT4/5: Neurotrophin-4/5
OCN: Osteocalcin
p75NTR: P75 neurotrophic factor receptor
PDGF-BB: Platelet-derived growth factor type BB
PEG: Polyethyleneglycol
PI3K: Phosphatidylinositide 3-kinase
PWH: Piezoelectric WH
REDV: Arg-Gly-Asp-Val
rhBMP-7: Recombinant human BMP-7
RNAs: Ribonucleic acids
Runx2: Runt-related transcription factor 2
SCs: Schwann cells
Sema: Semaphorin
siRNA: Small interfering RNA
SLIT3: Slit guidance ligand 3
TrkA: Tyrosine kinase receptor A
VEGF: Vascular endothelial growth factor
WH: Whitlockite
YIGSR: Tyr-Lle-Gly-Ser-Arg
